# An Integrated Strategy for Investigating Antioxidants from *Ribes himalense* Royle ex Decne and Their Potential Target Proteins

**DOI:** 10.3390/antiox12040835

**Published:** 2023-03-29

**Authors:** Chuang Liu, Yuqing Lei, Youyi Liu, Jingrou Guo, Xingyi Chen, Yifei Tang, Jun Dang, Minchen Wu

**Affiliations:** 1 Wuxi School of Medicine, Jiangnan University, Wuxi 214122, China; 2 School of Biotechnology, Jiangnan University, Wuxi 214122, China; 3 Key Laboratory of Tibetan Medicine Research, Northwest Institute of Plateau Biology, Chinese Academy of Sciences, Xining 810001, China

**Keywords:** antioxidants, molecular docking, natural products, online HPLC-1,1-diphenyl-2-picrylhydrazyl, *Ribes himalense*

## Abstract

Natural products have been used extensively around the world for many years as therapeutic, prophylactic, and health-promotive agents. *Ribes himalense* Royle ex Decne, a plant used in traditional Tibetan medicine, has been demonstrated to have significant antioxidant and anti-inflammatory properties. However, the material basis of its medicinal effects has not been sufficiently explored. In this study, we established an integrated strategy by online HPLC-1,1-diphenyl-2-picrylhydrazyl, medium-pressure liquid chromatography, and HPLC to achieve online detection and separation of antioxidants in *Ribes himalense* extracts. Finally, four antioxidants with quercetin as the parent nucleus were obtained, namely, Quercetin-3-O-β-D-glucopyranoside-7-O-α-L-rhamnopyranoside, Quercetin-3-O-β-D-xylopyranosyl(1-2)-β-D-glucopyranoside, Quercetin-3-O-β-D-glucopyranoside, and Quercetin-3-O-β-D-galactoside. Until now, the four antioxidants in *Ribes himalense* have not been reported in other literatures. Meanwhile, the free-radical-scavenging ability of them was evaluated by DPPH assay, and potential antioxidant target proteins were explored using molecular docking. In conclusion, this research provides insights into the active compounds in *Ribes himalense* which will facilitate the advancement of deeper studies on it. Moreover, such an integrated chromatographic strategy could be a strong driver for more efficient and scientific use of other natural products in the food and pharmaceutical industries.

## 1. Introduction

The treasures of the Chinese medical system, namely, traditional Chinese medicines, have been utilized for thousands of years [1]. However, intricate formulations, unclear active ingredients, and other persistent problems have restrained their widespread promotion worldwide [2]. *Ribes himalense* Royle ex Decne (*R. himalense*) is a diminutive woody plant that harbors abundant resources for both medicinal and culinary purposes [3]. It is mainly distributed in Sichuan, Qinghai, and Tibet of China. In Tibetan and Uyghur medicine systems, *R. himalense* is used to treat oxidative stress, arthritis, hepatitis, and various vascular diseases [4,5,6]. The fruit of *R. himalense* is a berry and is often used by folk to produce juices, jams, wines, preserves, and other highly nutritious by-products. In addition, its stems and leaves are commonly consumed as tea, which can achieve the effects of clearing heat, detoxifying, and moisturizing organs. However, due to the high growing altitude and lack of suitable analytical techniques, the nutritional value and health benefits of *R. himalense* are not supported by strong scientific evidence, which in turn hinders the widespread application of this plant in the modern food and pharmaceutical industries.

Elucidating the chemical composition of traditional Chinese medicines has been the focus of researchers to enable their widespread utilization globally [7]. The isolation of high-purity compounds in traditional Chinese medicines is not only beneficial to their modern pharmacological studies, but also key to the in-depth exploration of drug–disease interaction mechanisms [8,9]. At present, numerous techniques have been developed based on the chromatography concept to analyze and separate chemical components in natural products, including as gas chromatography, liquid chromatography, and high-speed counter-current chromatography, among which liquid chromatography is the most widely used [10,11,12,13]. Regarding liquid chromatography, the separation of natural products’ chemical constituents often involves a combination of medium-pressure liquid chromatography (MPLC) and HPLC, which is determined by its complexity. MPLC simplifies and visualizes the pretreatment of crude samples, and then HPLC is responsible for preparing high-purity compounds [14,15].

Researchers have demonstrated through numerous experiments that oxidative stress can disrupt the normal functioning of organisms, thereby inducing a range of diseases such as aging, inflammation, immune disorders, and tumors [16,17,18]. Antioxidant supplementation is particularly important in order to improve the state of oxidative stress in the organism and can also have a positive effect on related diseases. Based on this, the discovery, purification, and application of antioxidants have become hot research topics nowadays. 1,1-diphenyl-2-picrylhydrazyl (DPPH) is a stable nitrogen-centered free radical that can be combined with HPLC to form a post-column derivatization system, which has the potential to explore the antioxidant activity of compounds at the chromatographic level [19]. Compared with traditional methods for determining antioxidant activity, the online HPLC-DPPH technique, which has developed in recent years, is characterized by its simplicity of operation and high reproducibility. It enables rapid online identification of antioxidant components in natural products at the chromatographic level, thereby guiding subsequent separation work. Currently, online HPLC-DPPH has detected a large number of antioxidants from a variety of foods and herbal medicines [20,21].

Molecular docking is a cost-effective and time-efficient computer simulation technique used to predict the affinity of small molecules for their target proteins. In this technique, a small molecule (labeled ligand) is docked into the binding site of a protein (labeled receptor), by continuously changing the spatial conformation to predict the optimal binding mode and binding site of the ligand–receptor complex [22]. This technique is widely used in drug discovery for lead compound screening, lead compound optimization, and prediction of ligand–protein interactions [23,24]. Frankly, its limitations in terms of computer theoretical simulation often require further experimental validation. Nevertheless, due to its high throughput and predictability, a multitude of scientists still consider it a powerful tool to drive the development of related research fields [25].

Considering the strong medicinal background and the limited chemical composition studies of *R. himalense*, we conducted a complete work combining online HPLC-DPPH, and MPLC with HPLC, to recognize and isolate antioxidants in *R. himalense*. This work exclusively relied on chromatographic means, with the aim of achieving high efficiency, reproducibility, and stability. We also measured the antioxidant capacity of four isolated compounds by the DPPH method to verify the reliability of online HPLC-DPPH. In addition, to further explore the biological activities of these compounds as well as potential antioxidant target proteins, nine related proteins were selected for molecular docking with them to elucidate their binding affinity. The conclusions obtained may offer a theoretical underpinning for the in-depth development of the plant as food and pharmaceutical products.

## 2. Materials and Methods

### 2.1. Equipment

The ESI-MS data of the isolated compound were acquired using Waters ZQ 2000 (Waters, Milford, MA, USA). The NMR data were collected using Bruker Avance AV NEO 600 MHz (Bruker, Karlsruhe, Germany), and the results were analyzed and visualized via MestReNova 12.0.4 (Mestrelab Research, La Coruna, Galicia, Spain). The basic formulation of the online HPLC-DPPH system was as follows: an 18.0 m reaction coil was used to connect the LC-16 (first HPLC) and LC-10AD (second HPLC) instruments, and each chromatograph was equipped as standard with a UV–Vis detector, two binary gradient pumps, an automatic degasser, and a workstation for Shimadzu (Shimadzu, Kyoto, Japan). The samples were separated on a preparative HPLC system from Jiangsu Hanbang Technology Co., Ltd., Huaian, China.

### 2.2. Chemicals and Reagents

The following chromatographic columns and separation materials were utilized in this paper. The filler in the polyamide (100–200 mesh) medium pressure column (49 mm × 460 mm) was purchased from Jiangsu Changfeng Chemical, China, and its particle size is between 100 and 200 mesh. Spherical C18 (50 µm particle size) was purchased from SiliCycle in Canada and employed in a medium pressure column (50 mm × 500 mm). A Reprosil-Pur C18 AQ analytical column (4.6 × 250 mm, 5 μm) was supplied by Maisch Corporation (Munich, Germany). A Click XIon analytical column (4.6 × 250 mm, 5 μm) and Click XIon preparative column (20 × 250 mm, 5 μm) were obtained from Acchrom Technologies, Beijing, China. DPPH was obtained from Sigma-Aldrich (Steinheim, Germany).

Ethanol and methanol of analytical grade were purchased from Kelon Chemical Reagent Factory in Chengdu, China. Acetonitrile (ACN) and methanol of HPLC grade were purchased from Xinlanjing Chemical Industry in Yunnan, China. Ultrapure water resistivity of 18.2 MΩ × cm, was from a Moore water filtration device (Chongqing, China).

### 2.3. Extraction and Pretreatment of Crude Sample

*Ribes himalense* was discovered in Xiewu Town, Yushu Tibetan Autonomous Prefecture, Qinghai Province, China and validated by Prof. Lijuan Mei of the Northwest Institute of Plateau Biology, Chinese Academy of Sciences. A sample (No. 0342650) is preserved for review in the Qinghai–Tibetan Platea Museum of Biology, Chinese Academy of Sciences [3]. Fresh stems and leaves of this plant were cleaned with distilled water, air-dried, and crushed to obtain 300 g powder before being mixed with 8 L of 95% ethanol. After 12 h, the extraction solution was filtered and poured out. Then, 8 L of fresh ethanol was added again to the 300 g powder. This extraction procedure was repeated three times, resulting in a total of 24 L of combined extract solution. A rotary evaporator was used for vacuum concentration, with a temperature of 40 °C and speed of 100 rpm. The concentration was halted once the extract solution volume had decreased to roughly 0.5 L, then dried in an oven at 60 °C with 105 g of polyamide mixed in.

The MPLC pretreatment followed the literature [26], with minor modifications. A small empty chromatography tower (49 × 100 mm) was used as the carrier for dry-loading the polyamide–extract mixture (130.3 g). The chromatography tower was then connected to a polyamide medium pressure column and MPLC for the pretreatment. H_2_O and ACN comprised the mobile phase, and polyamide–extract mixture was linearly eluted by setting the chromatographic conditions of 0–100 min, 0–100% ACN, with a flow rate of 57 mL/min. The fractions were recovered based on the absorption value of sample at 254 nm. A total of 4 fractions (labeled Fr1, Fr2, Fr3, and Fr4) were recovered after 13 separations. The weights of Fr1, Fr2, Fr3, and Fr4 were as follows: 8.7 g, 2.9 g, 6.3 g, and 1.4 g.

Fr3 was further pretreated by Spherical C18-MPLC. A total of 6.3 g was completely dissolved in 80 mL of methanol, and the sample was linearly eluted by a mobile phase consisting of H_2_O and ACN, with the program set to 0–80 min, 15–100% ACN, 57 mL/min. Similarly, the fractions were recovered based on the absorption value of the sample at 254 nm. Finally, after 8 iterations of elution, Fr3-1 (1.1 g) was obtained.

In order to visualize the effect of the MPLC pretreatment, the fractions obtained from each pretreatment recovery were analyzed on the Reprosil-Pur C18 AQ analytical column. For polyamide-MPLC, the mobile phase composed of H_2_O and ACN was used with the following linear gradient elution parameters: 0–60 min, 5–95% ACN. As for Spherical C18-MPLC, a linear elution procedure of 0–30 min, 5–50% ACN was performed for each fraction. All chromatographic data were observed at 254 nm.

### 2.4. Chromatographic Analysis of Target Fraction

The target fraction Fr3-1, completely dissolved in 7 mL methanol, was first analyzed by Reprosil-Pur C18 AQ analytical column. The binary mobile phases were H_2_O and ACN, respectively. Gradient elution was carried out for 30 min, during which the concentration of ACN ranged from 5% to 80%. Simultaneously, a Click XIon analytical column was also used to analyze Fr3-1. A mobile phase gradient consisting of ACN and H_2_O was eluted over 60 min with ACN concentrations ranging from 87% to 97%. All above processes of elution were tracked at 254 nm.

### 2.5. Online Identification and Directional Separation of Active Peaks by Hydrophilic-HPLC

The online HPLC-DPPH activity screening system was constructed following the method of Dang [27], with the DPPH solution used as the mobile phase for the second HPLC. The Click XIon analytical column was used to analyze Fr3-1, with the same chromatographic and elution conditions as described in Section 2.4. In addition, DPPH was prepared using ethanol at a concentration of 50 μg/mL, and the flow rate was 0.8 mL/min. Based on the inverted peaks at 517 nm, the data of active peaks in the sample were acquired.

The directional separation of antioxidants in Fr3-1 was completed by a Click XIon preparative column, and the chromatographic conditions for sample analysis were directly linearly amplified to the preparative level. The injection volume was maintained at 0.5 mL, and the flow rate was set to 19 mL/min. After 14 cycles of preparation, 31.7 mg of Fr3-1-1, 21.7 mg of Fr3-1-2, 35.8 mg of Fr3-1-3, and 79.8 mg of Fr3-1-4 were obtained.

### 2.6. Evaluation of Purity and Activity of Antioxidative Flavonoids

The isolated compounds were evaluated for purity as well as free-radical-scavenging activity via online HPLC-DPPH. The samples were eluted with a linear gradient using a mobile phase combination of H_2_O and ACN, with the elution program set to 5–55% ACN, 1 mL/min for 30 min. Moreover, the concentration and flow rate of DPPH solution were kept consistent with the conditions in Section 2.5. Chromatographic data of each compound were retrieved at 254 nm, and their antioxidant activities were evaluated at 517 nm.

The antioxidant activity of 4 isolated compounds was further evaluated using the DPPH method, following a modified protocol based on the literature [28]. Initially, the compounds were prepared as sample solutions at 6 different concentrations (0.1, 1, 10, 50, 100, and 500 μg/mL) and 30 μL of each concentration was pipetted into a 96-well plate, then mixed with 70 μL of DPPH (0.25 mg/mL) solution and incubated for 30 min in the dark. The absorbance of each sample solution was measured at 517 nm by a microplate reader. The experiment was repeated three times to ensure the reliability of results. The following equation was used to calculate the antioxidant capacity of each compound:

Inhibitory activity (%) = [1 − (A_0_ − A_1_)/A_2_] × 100%

The absorbances of the sample solution, blank, and control were represented by A_0_, A_1_, and A_2_, respectively. The IC_50_ value of each compound was finally obtained by fitting the inhibitory activity curves.

### 2.7. Molecular Docking Study

Molecular docking experiments required the 3D structure of the ligand as well as crystal structure of receptor. For this experiment, 3D structures of antioxidants were drawn by ChemBio3D software and exported as mol2 files. Meanwhile, the crystal structures of 9 related proteins were retrieved from the RCSB Protein Data Bank inhttps://www.rcsb.org/ (accessed on 15/12/2022) as follows: Acetylcholinesterase (AChE, PDB ID: 1OCE), Cytochrome P450 2C9 (CYP2C9, PDB ID: 1OG5), Nitric oxide synthase (iNOS, PDB ID: 1M8D), NADPH-oxidase (PDB ID: 2CDU), NF-E2-related factor 2 (Nrf2, PDB ID: 4IQK), Superoxide dismutase (SOD, PDB ID: 1CBJ), Tumor necrosis factor α (TNF-α, PDB ID: 2AZ5), Xanthine oxidase (XOD, PDB ID: 3NRZ), and α-glucosidase (PDB ID: 3A4A). After preparing these ligand and receptor files, the receptor proteins were processed by PyMOL and AutoDock software, including dehydration, removal of ligands, and addition of charges. Then, the AutoGrid program framed the active pocket coordinates of the receptor proteins, and the results of 100 peptide conformations in which the ligands were able to bind to the proteins were obtained by AutoDock software. Detailed information about proteins and molecular docking was listed in Table 1. Finally, PyMOL was used to analyze and visualize the docking results with the lowest binding energy.

### 2.8. Statistical Analysis

The statistics of the data were realized by SPSS 20.0 (SPSS, Chicago, IL, USA) software, and the values were expressed as mean ± SD. In addition, the inhibitory activity curves of each compound were plotted by Prism 8.0.

## 3. Results and Discussion

### 3.1. Sample Pretreatment with MPLC

**Figure 1:**
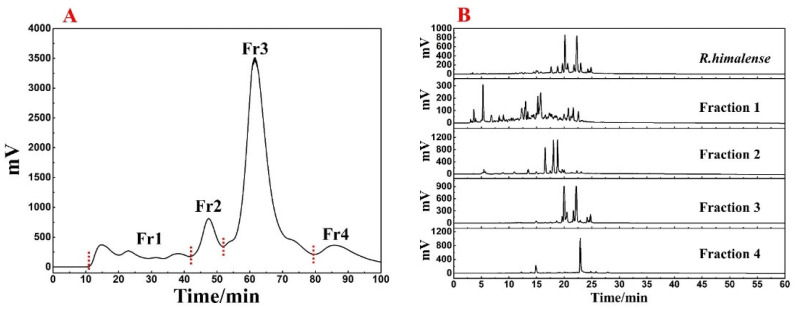
(**A**) polyamide-MPLC separation chromatogram of *R. himalense* extract-polyamide mixture. (**B**) Comparative chromatogram of the crude sample and each fraction after pretreatment.

The utilization of ethanol as an extraction solvent is an effective approach that can minimize environmental pollution as well as preserve the diversity of *R. himalense* extract. Finally, a total of 25.3 g extract was recovered from 300 g of stems and leaves, with a satisfactory yield (8.43%). The polyamide-MPLC pretreatment of *R. himalense* extract is presented in Figure 1A. It can be seen that the baseline separation between the four fractions was basically achieved. By further analysis of each fraction with crude sample (Figure 1B), it can be seen that there is no obvious overlap phenomenon between each fraction, and all components of the crude sample were included, indicating the excellent pretreatment effect.

**Figure 2:**
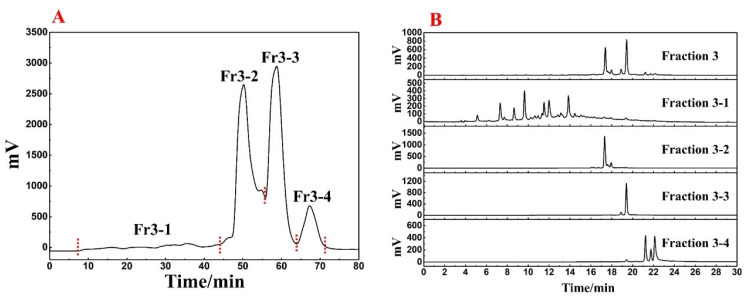
(**A**) Spherical C18-MPLC separation chromatogram of Fr3. (**B**) Comparative chromatogram of Fr3 and each fraction after pretreatment.

Based on Figure 1, it was clear that the main component of *R. himalense* was enriched in Fr3. Considering the sample amount and complexity of Fr3, a Spherical C18-MPLC with a smaller particle size was selected for its pretreatment. Figure 2A showed the pretreatment chromatogram of Fr3, where the same elution procedure was repeated eight times to recover a total of four fractions (labeled Fr3-1, Fr3-2, Fr3-3, and Fr3-4). As before, the four recovered fractions were analyzed together with Fr3 (Figure 2B), and it was found that there was no overlap between the four fractions, some trace ingredients were efficiently enriched in Fr3-1, while the obvious chromatographic peaks in Fr3 were effectively enriched in the other fractions (Fr3-2 and Fr3-3), respectively.

Summarizing the above, polyamide-MPLC with Spherical C18-MPLC was excellently competent for the pretreatment of *R.himalense*, achieving the purpose of online visual separation, removing non-target components, and simplifying subsequent separation. As the target fraction for investigation in this paper, 1.1 g of Fr3-1 was fully dissolved in 7 mL of methanol to illustrate the qualitative identification and targeted separation of antioxidant active ingredients in *R.himalense*.

### 3.2. Analysis and Activity Peaks Identification of Fr3-1

**Figure 3:**
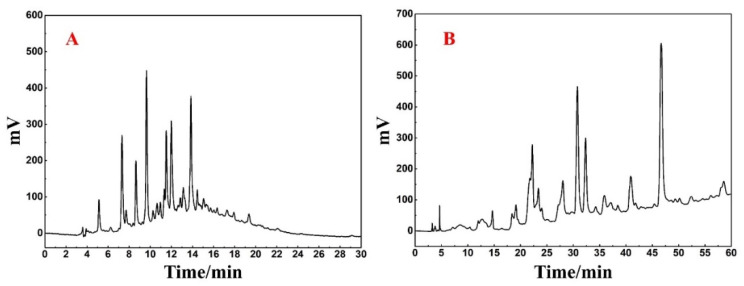
(**A**) Analysis chromatogram of Fr3-1 on Reprosil-Pur C18 AQ analytical column. (**B**) Analysis chromatogram of Fr3-1 on Click XIon analytical column.

Upon analysis of Fr3-1 using the Reprosil-Pur C18 AQ analytical column (Figure 3A), it was revealed that a large peak capacity and poor resolution with peak retention times mostly centered around 6–16 min. Such analytical conditions may cause sample loss and inefficiency if directly linearly scaled up to preparative level. At the same time, the Click XIon column with large selectivity difference from the Spherical C18 column was used for the re-analysis of Fr3-1. It can be seen from Figure 3B that Fr3-1 exhibited good peak resolution on the hydrophilic chromatographic column, and visual separation between multiple peaks was basically possible. Based on the above discussion and the complexity of Fr3-1, it was finally determined that Click XIon column was used for the high-pressure separation of Fr3-1 to ensure the highest possible preparation efficiency as well as the recovery of high-purity antioxidants.

**Figure 4:**
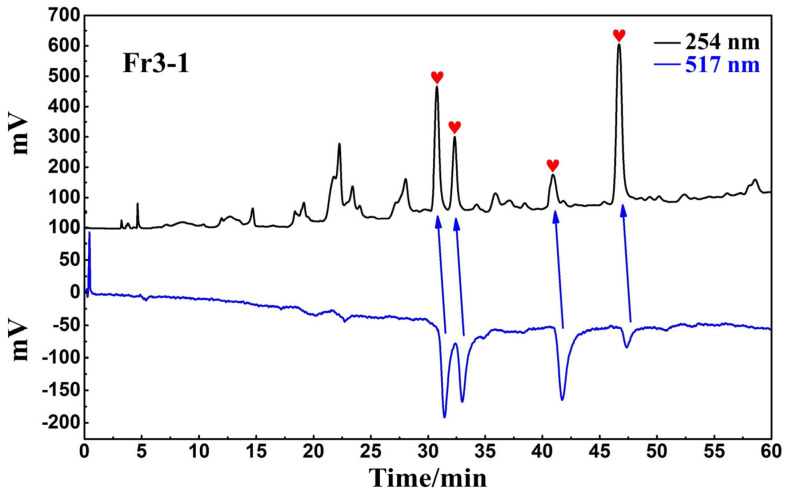
Online HPLC-DPPH activity screening chromatogram of Fr3-1 on a Click XIon analytical column.

The antioxidant peaks in Fr3-1 were recognized using a Click XIon analytical column in the online HPLC-DPPH assay. The chromatogram of the online recognition is shown in Figure 4, where a total of four distinct activity peaks were detected (labeled as red hearts), distributed over retention times between 30 and 50 min.

### 3.3. Directed Separation of Antioxidants from Fr3-1

**Figure 5:**
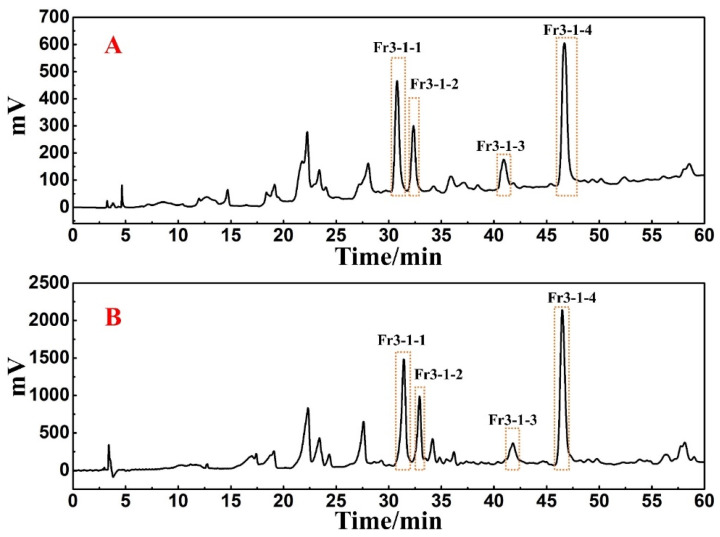
(**A**) Analysis chromatogram of Fr3-1 on a Click XIon analytical column. (**B**) Preparative chromatogram of Fr3-1 on a Click XIon preparative column.

A Click XIon preparative column was used to isolate the antioxidants in Fr3-1. The flow rate was set to 19 mL/min and the loading volume was determined to be 0.5 mL, while other conditions were kept constant. The preparative chromatogram was shown in Figure 5B. The retention times of the four target peaks were found to be consistent when comparing the preparative chromatogram with the analytical chromatogram (Figure 5A). Moreover, 0.5 mL loading volume each time can still ensure the visual separation of chromatographic peaks without affecting the recovery of high-purity antioxidants, as well as reducing the separation workload. Finally, Fr3-1-1, Fr3-1-2, Fr3-1-3, and Fr3-1-4 were obtained after 14 separations, and their weights were 31.7 mg, 21.7 mg, 35.8 mg, and 79.8 mg, respectively.

### 3.4. Purity, Structural Characterization, and Activity of the Isolated Antioxidants

**Figure 6:**
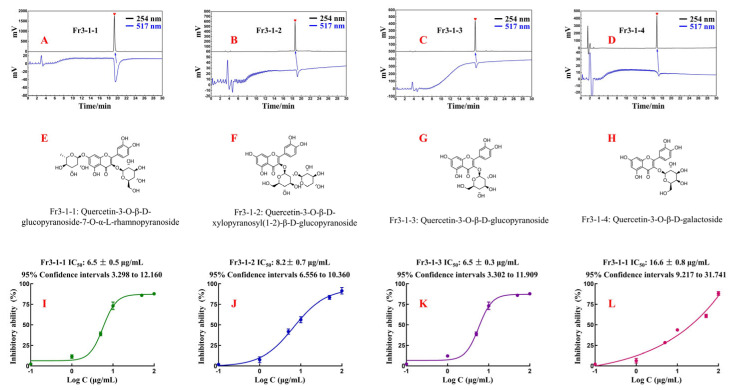
(**A**–**D**) Activity verification chromatograms of Fr3-1-1, Fr3-1-2, Fr3-1-3, and Fr3-1-4. (**E**–**H**) Chemical structures and names of Fr3-1-1, Fr3-1-2, Fr3-1-3, and Fr3-1-4. (**I**–**L**) DPPH clearance curves of Fr3-1-1, Fr3-1-2, Fr3-1-3, and Fr3-1-4.

Fr3-1-1, Fr3-1-2, Fr3-1-3, and Fr3-1-4 were analyzed again using an online HPLC-DPPH on a Reprosil-Pur C18 AQ analytical column. The results are shown in Figure 6A–D. All four antioxidants displayed satisfactory levels of purity and antioxidant activity, thereby attesting to the reliability and effectiveness of our previous work. The displacement information of the hydrogen and carbon atoms of each compound, as well as their fragment ion peak, were analyzed and compared with the literature. Finally, the chemical structures of four antioxidants were identified (Figure 6E–H). More detailed spectral information was presented in the Supplementary Material (Figures S1–S12).

Fr3-1-1 (Quercetin-3-O-β-D-glucopyranoside-7-O-α-L-rhamnopyranoside, 31.7 mg, yellow powder, ESI-MS *m*/*z* 609.31 [M-H]^-^, calc. For C_27_H_30_O_16_, *m/z* 601.15); ^1^H-NMR (600 MHz, CD_3_OD): δ 7.69 (1H, d, *J* = 2.1 Hz, H-2′), 7.57 (1H, dd, *J* = 8.5, 2.1 Hz, H-6′), 6.86 (1H, d, *J* = 8.5 Hz, H-5′), 6.36 (1H, d, *J* = 2.1 Hz, H-8), 6.17 (1H, d, *J* = 2.1 Hz, H-6), 5.74 (1H, d, *J* = 7.7 Hz, H-1″), 5.20 (1H, d, *J* = 1.5 Hz, H-1′′′), 4.03~3.48 (10H, m, sugar protons), 0.91 (3H, d, *J* = 6.2 Hz, H-6′′′); ^13^C-NMR (151 MHz, CD_3_OD): δ 179.4 (C-4), 165.6 (C-7), 163.2 (C-5), 158.4 (C-9), 158.2 (C-2), 149.6 (C-4′), 145.9 (C-3′), 134.6 (C-3), 123.3 (C-1′), 123.0 (C-6′), 117.3 (C-5′), 116.1 (C-2′), 105.9 (C-1″), 102.6 (C-1′′′), 100.8 (C-10), 99.6 (C-6), 94.4 (C-8), 77.6 (C-5″), 77.2 (C-3″), 75.7 (C-2″), 74.1 (C-4″) 72.4 (C-3″), 72.3 (C-2′′′), 70.9 (C-5′′′), 69.8 (C-4″), 62.1 (C-6″), 17.4 (C-6′′′). These data agree with the information reported in the literature [29,30] on the spectra of Quercetin-3-O-β-D-glucopyranoside-7-O-α-L-rhamnopyranoside.

Fr3-1-2 (Quercetin-3-O-β-d-xylopyranosyl(1-2)-β-d-glucopyranoside, 21.7 mg, light yellow powder, ESI-MS *m*/*z* 595.30 [M-H]^-^, calc. For C_26_H_28_O_16_, *m*/*z* 596.14); ^1^H-NMR (600 MHz, CD_3_OD): δ 7.63 (1H, dd, *J* = 8.5, 2.2 Hz, H-6′), 7.62 (1H, d, *J* = 2.2 Hz, H-2′), 6.86 (1H, d, *J* = 8.5 Hz, H-5′), 6.37 (1H, d, *J* = 2.1 Hz, H-8), 6.18(1H, d, *J* = 2.1 Hz, H-6), 5.50 (1H, d, *J* = 7.6 Hz, H-1″), 4.75 (1H, d, *J* = 7.1 Hz, H-1′′′), 3.93~3.20 (11H, m, sugar protons); ^13^C-NMR (151 MHz, CD_3_OD): δ 179.6 (C-4), 165.8 (C-7), 163.2 (C-5), 158.4 (C-9), 158.3 (C-2), 149.7 (C-4′), 146.1 (C-3′), 135.2 (C-3), 123.4 (C-1′), 123.2 (C-6′), 117.3 (C-5′), 116.1 (C-2′), 105.8 (C-1′′′), 105.4 (C-1″), 100.8 (C-10), 99.7 (C-6), 94.5 (C-8), 82.3 (C-5″), 78.4 (C-2″), 78.3 (C-35′) 77.0 (C-3′′′), 74.9 (C-2′′′), 71.1 (C-4″), 71.0 (C-4′′′), 66.6 (C-5′′′), 62.4 (C-6″). These data agreed with the information reported in the literature [31] on the spectra of Quercetin-3-O-β-d-xylopyranosyl(1-2)-β-d-glucopyranoside.

Fr3-1-3 (Quercetin-3-O-β-d-glucopyranoside, 35.8 mg, light yellow powder, ESI-MS *m*/*z* 487.23 [M+Na]^+^, calc. For C_21_H_20_O_12_, *m*/*z* 464.10); ^1^H-NMR (600 MHz, CD_3_OD): δ 7.72 (1H, d, *J* = 2.2 Hz, H-2′), 7.61 (1H, dd, *J* = 8.5, 2.2 Hz, H-6′), 6.89 (1H, d, *J* = 8.5 Hz, H-5′), 6.42 (1H, d, *J* = 2.1 Hz, H-8), 6.22 (1H, d, *J* = 2.1 Hz, H-6), 5.27 (1H, d, *J* = 7.7 Hz, H-1″), 3.74~3.26 (6H, m, sugar protons); ^13^C-NMR (151 MHz, CD_3_OD): δ 179.5 (C-4), 166.0 (C-7), 163.1 (C-5), 159.0 (C-9), 158.5 (C-4′), 149.9 (C-2), 145.9 (C-3′), 135.6 (C-3), 123.3 (C-6′), 123.1 (C-1′), 117.5 (C-5′), 116.0 (C-2′), 105.7 (C-10), 104.3 (C-1″), 99.9 (C-6), 94.7 (C-8), 78.4 (C-5″), 78.1 (C-3″), 75.7 (C-2″), 71.2 (C-4″), 62.6(C-6″). These data agreed with the information reported in the literature [32] on the spectra of Quercetin-3-O-β-d-glucopyranoside.

Fr3-1-4 (Quercetin-3-O-β-d-galactoside, 79.8 mg, light yellow powder, ESI-MS *m*/*z* 487.22 [M+Na]^+^, calc. For C_21_H_20_O_12_, *m*/*z* 464.10); ^1^H-NMR (600 MHz, CD_3_OD): δ 7.86 (1H, d, *J* = 2.2 Hz, H-2′), 7.60 (1H, dd, *J* = 8.5, 2.2 Hz, H-6′), 6.88 (1H, d, *J* = 8.5 Hz, H-5′), 6.42 (1H, d, *J* = 2.1 Hz, H-8), 6.22 (1H, d, *J* = 2.1 Hz, H-6), 5.18 (1H, d, *J* = 7.8 Hz, H-1″), 3.87~3.48 (6H, m, sugar protons); ^13^C-NMR (151 MHz, CD_3_OD): δ 179.6 (C-4), 166.1 (C-7), 163.0 (C-5), 158.8 (C-9), 158.5 (C-2), 150.0 (C-4′), 145.8 (C-3′), 135.8 (C-3), 122.9 (C-1′, 6′), 117.8 (C-5′), 116.1 (C-2′), 105.7 (C-1″), 105.4 (C-10), 99.9 (C-6), 94.7 (C-8), 77.2 (C-2″), 75.1 (C-5″), 73.2 (C-3″), 70.0 (C-4″), 61.9 (C-6″). These data agreed with the information reported in the literature [33] on the spectra of Quercetin-3-O-β-d-galactoside.

The inhibitory activity curves of four isolated compounds (Figure 6I–L) were generated using the method outlined in 2.6. The IC_50_ value represents the concentration required for 50% inhibitory activity of the sample. Based on this, the IC_50_ values of the four compounds were accurately calculated to evaluate their antioxidant activity. Specifically, the IC_50_ value of Fr3-1-1 was 6.5 ± 0.5 μg/mL, the IC_50_ value of Fr3-1-2 was 8.2 ± 0.7 μg/mL, the IC_50_ value of Fr3-1-3 was 6.5 ± 0.3 μg/mL, and the IC_50_ value of Fr3-1-4 was 16.6 ± 0.8 μg/mL. Theoretically, the lower the IC_50_ value, the better the antioxidant activity. All these compounds are flavonoids with quercetin as the parent nucleus. For Fr3-1-3 and Fr3-1-4, the antioxidant capacity was nearly twice as strong when the C-3 position substituent was replaced from galactoside to glucopyranoside. As for Fr3-1-1 and Fr3-1-3, when the hydroxyl group at the C-7 position was replaced by rhamnopyranoside, the IC_50_ value remained basically unchanged. Overall, Fr3-1-1, Fr3-1-2, Fr3-1-3, and Fr3-1-4 showed excellent antioxidant capacity in the DPPH assay, which also confirmed the accuracy and stability of the online HPLC-DPPH. The popularity of online HPLC-DPPH in the fields of food and pharmaceuticals is constantly increasing [20,21], and our experimental results actively promote its evolution.

### 3.5. Molecular Docking Study of Related Target Proteins and Antioxidants

**Figure 7:**
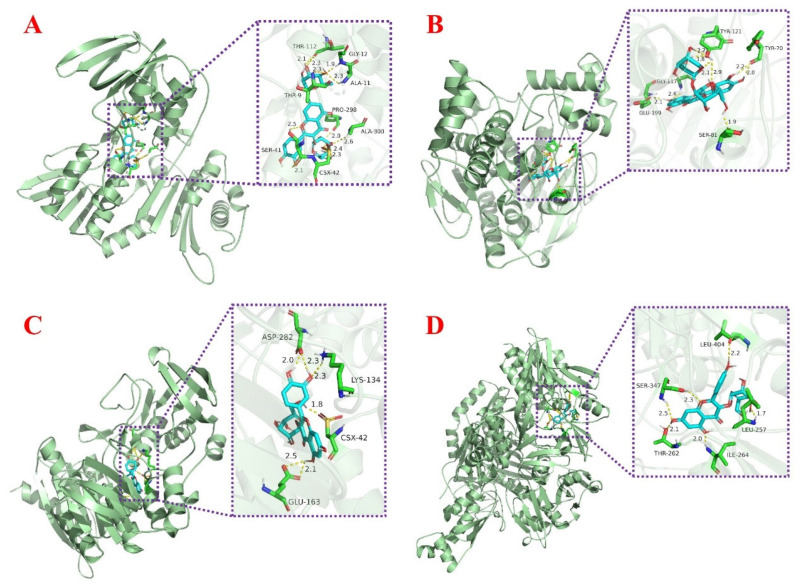
(**A**) Representation of molecular interaction between NADPH-oxidase and Fr3-1-1 based on molecular docking. (**B**) Representation of molecular interaction between AChE and Fr3-1-2 based on molecular docking. (**C**) Representation of molecular interaction between NADPH-oxidase and Fr3-1-3 based on molecular docking. (**D**) Representation of molecular interaction between XOD and Fr3-1-4 based on molecular docking.

The regulation of oxidative stress in organisms cannot be achieved without the involvement of proteins. In this study, nine proteins related to oxidative stress were selected for molecular docking in order to further investigate the possible binding target proteins of the isolated compounds, including AChE, CYP2C9, iNOS, NADPH-oxidase, Nrf2, SOD, TNF-α, XOD, and α-glucosidase. The final docking scores obtained were presented in the form of a heat map in Table 2, where the color trend (green–yellow–red) indicates the variation in docking values. Red color indicates the lowest docking value, while green color indicates the highest docking value. This visual display easily revealed a trend of compounds acting as potential inhibitors of the target protein. Figure 7 showed the docking posture of each compound with its protein having the lowest binding energy. More docking posture were shown in Figures S13–S16. As a key enzyme in biological nerve conduction, AChE is involved in the development of cells and nerves in the body and promotes the normal transmission of nerve signals in the organism. The docking results showed that Fr3-1-2 exhibited the best binding energy to AChE, mainly through the active pocket built by the amino acid residues Glu-199, Gly-117, Ser-81, Tyr-70, and Tyr-121. XOD belongs to aerobic dehydrogenase, which can catalyze hypoxanthine to generate uric acid and hydrogen peroxide (Figure 7B). Meanwhile, XOD is one of the important enzymes in nucleic acid metabolism in vivo and is also involved in iron uptake and transport in vivo. In the present experiment, Fr3-1-4 showed stronger binding ability (−8.56 kcal/mol) to XOD. Specifically, it functions by establishing hydrogen bonds through Leu-404, Leu-257, Ser-347, Thr-262, and Ile-264 amino acid residues (Figure 7D). As for Fr3-1-1 and Fr3-1-3, the lowest docking energy with them was NADPH-oxidase, and the detailed docking posture was shown in Figure 7A and C. NADPH-oxidase can catalyze the body to generate a large amount of reactive oxygen species, which plays an important role in pathological processes such as inflammation, tumorigenesis, and fibrosis. The good combination of these two compounds with the protein indicates that they may also play an active role in the above pathological process.

Throughout all the docking results, four compounds exhibited strong binding energy to the nine selected target proteins, which provided a reasonable explanation for the ability of these compounds to exert antioxidant effects, and also provided ideas to explore their other biological activities. However, it is essential to note that rigorous and thorough experiments are always needed to further demonstrate how these compounds perform their antioxidant functions in the organism.

## 4. Conclusions

This present study was carried out to establish a rapid and efficient chromatographic method for the identification and separation of antioxidants in *R. himalense*. Through a sequential combination of MPLC, online HPLC-DPPH, and hydrophilic-HPLC, a total of four antioxidants with quercetin as the parent nucleus were obtained, namely, Quercetin-3-O-β-d-glucopyranoside-7-O-α-L-rhamnopyranoside, Quercetin-3-O-β-d-xylopyranosyl(1-2)-β-d-glucopyranoside, Quercetin-3-O-β-d-glucopyranoside, and Quercetin-3-O-β-d-galactoside, which were the first isolations from this plant. In addition. The DPPH method demonstrated that they possess excellent free-radical-scavenging ability, while molecular docking further explored their antioxidant mechanisms, and also provided a direction for the exploration of their other biological activities. In conclusion, antioxidants in *R. himalense* were investigated at the chromatographic level, with visual monitoring and online separation throughout the process with high reliability and good reproducibility. At the same time, the docking of the isolated antioxidants with the potential proteins rapidly explored their biological activities and mechanisms from a theoretical point of view. This complete research strategy provides a basis for a more rational and scientific use of *R. himalense*, and is also valuable for the development of other foods and pharmaceutical products.

## Figures and Tables

**Table 1:** Molecular docking parameters and protein targets.

Protein	PDB ID	Number of Points	Center Grid Box	Spacing	GA Runs
**AChE**	1OCE	X_dimension = 50 Y_dimension = 52 Z_dimension = 46	X_center = 3.569 Y_center = 63.317 Z_center = 63.91	0.375	100
**CYP2C9**	1OG5	X_dimension = 70 Y_dimension = 62 Z_dimension = 92	X_center = −51.194 Y_center = 50.676 Z_center = 24.829	0.375	100
**iNOS**	1M8D	X_dimension = 80 Y_dimension = 66 Z_dimension = 64	X_center = 125.463 Y_center = 105.408 Z_center = 36.29	0.375	100
**NADPH-oxidase**	2CDU	X_dimension = 98 Y_dimension = 60 Z_dimension = 58	X_center = 15.786 Y_center = 8.025 Z_center = 50.432	0.375	100
**Nrf2**	4IQK	X_dimension = 30 Y_dimension = 44 Z_dimension = 48	X_center = −44.568 Y_center = 1.471 Z_center = -16.41	0.375	100
**SOD**	1CBJ	X_dimension = 90 Y_dimension = 94 Z_dimension = 82	X_center = 8.655 Y_center = 24.873 Z_center = 42.192	0.375	100
**TNF-α**	2AZ5	X_dimension = 38 Y_dimension = 52 Z_dimension = 44	X_center = −23.465 Y_center = 71.695 Z_center = 35.798	0.469	100
**XOD**	3NRZ	X_dimension = 78 Y_dimension = 126 Z_dimension = 150	X_center = 89.328 Y_center = 0.287 Z_center = 39.243	0.375	100
**α-glucosidase**	3A4A	X_dimension = 72 Y_dimension = 50 Z_dimension = 78	X_center = 21.891 Y_center = −8.54 Z_center = 18.132	0.375	100

**Table 2:** Heat map of recorded docking scores of these ligands and protein targets.

	Ligand	Fr3-1-1	Fr3-1-2	Fr3-1-3	Fr3-1-4
Protein		Binding Free Energy kcal/mol			
**AChE**		−9.22	−8.94	−8.21	−8.16
**CYP2C9**		−6.03	−6.88	−6.15	−6.77
**iNOS**		−7.75	−8.24	−7.3	−7.9
**NADPH-oxidase**		−9.6	−8.25	−8.67	−8.25
**Nrf2**		−5.21	−5.36	−4.76	−4.85
**SOD**		−5.91	−8.08	−6.18	−6.08
**TNF-α**		−5.81	−5.15	−5.55	−5.94
**XOD**		−8.36	−6.28	−8.14	−8.56
**α-glucosidase**		−7.69	−7.76	−7.19	−7.48

Notes: AChE, Acetylcholinesterase; CYP2C9, Cytochrome P450 2C9; iNOS, Nitric oxide synthase; Nrf2, NF-E2-related factor 2; SOD, Superoxide dismutase; TNF-α, Tumor necrosis factor α; XOD, Xanthine oxidase. Color (green–yellow–red) represents the trend of docking values.

## Data Availability

Not applicable.

## Supplementary Materials

The following supporting information can be downloaded at: https://www.mdpi.com/article/10.3390/antiox12040835/s1, Figure S1: ESI-MS spectrum of Fr3-1-1. Figure S2: ^1^H NMR (600 MHz) data of Fr3-1-1 (in CD_3_OD). Figure S3: ^13^C NMR (151 MHz) data of Fr3-1-1 (in CD_3_OD). Figure S4: ESI-MS spectrum of Fr3-1-2. Figure S5: ^1^H NMR (600 MHz) data of Fr3-1-2 (in CD_3_OD). Figure S6: ^13^C NMR (151 MHz) data of Fr3-1-2 (in CD_3_OD). Figure S7: ESI-MS spectrum of Fr3-1-3. Figure S8: ^1^H NMR (600 MHz) data of Fr3-1-3 (in CD_3_OD). Figure S9: ^13^C NMR (151 MHz) data of Fr3-1-3 (in CD_3_OD). Figure S10: ESI-MS spectrum of Fr3-1-4. Figure S11: ^1^H NMR (600 MHz) data of Fr3-1-4 (in CD_3_OD). Figure S12: ^13^C NMR (151 MHz) data of Fr3-1-4 (in CD_3_OD). Figure S13: Interaction of Fr3-1-1 with active sites of nine potential target proteins. Figure S14: Interaction of Fr3-1-2 with active sites of nine potential target proteins. Figure S15: Interaction of Fr3-1-3 with active sites of nine potential target proteins. Figure S16: Interaction of Fr3-1-4 with active sites of nine potential target proteins.
