# Supplementary material for: An Integrated Strategy for Investigating Antioxidants from Ribes himalense Royle ex Decne and Their Potential Target Proteins
Source: Antioxidants (Basel). 2023 Mar 29;12(4):835. doi: 10.3390/antiox12040835 (PMC10135234; doi:10.3390/antiox12040835)
Supplement: Supplementary file 1 [file antioxidants-12-00835-s001.zip › antioxidants-2281084-supplementary.pdf]

Supporting Information

An integrated strategy for investigating antioxidants from *Ribes himalense* Royle ex Decne and their potential target proteins

Chuang Liu[a,b], Yuqing Lei[a,b], Youyi Liu[a], Jingrou Guo[a], Xingyi Chen[a], Yifei Tang[a], Jun Dang[c,*], Minchen Wu[a,*]

[a]Wuxi School of Medicine, Jiangnan University, Wuxi 214122, China

[b]School of Biotechnology, Jiangnan University, Wuxi 214122, China

[c]Key Laboratory of Tibetan Medicine Research, Northwest Institute of Plateau Biology, Chinese Academy of Sciences, Xining 810000, China

*__Corresponding authors__: Key Laboratory of Tibetan Medicine Research, Northwest Institute of Plateau Biology, Chinese Academy of Sciences, no. 23 Xinning Road, Xining, China.
Wuxi School of Medicine, Jiangnan University, no. 1800 Lihu Road, Wuxi, China.
E-mail: dangjun@nwipb.cas.cn (Jun Dang), biowmc@126.com (Minchen Wu).

Table of Contents

Figure S1. ESI-MS data of Fr3-1-1 3

Figure S2. ^{1}H NMR (600 MHz) data of Fr3-1-1 (in CD_3OD) 4

Figure S3. ^{13}C NMR (151 MHz) data of Fr3-1-1 (in CD_3OD) 5

Figure S4. ESI-MS data of Fr3-1-2 6

Figure S5. ^{1}H NMR (600 MHz) data of Fr3-1-2 (in CD_3OD) 7

Figure S6. ^{13}C NMR (151 MHz) data of Fr3-1-2 (in CD_3OD) 8

Figure S7. ESI-MS data of Fr3-1-3 9

Figure S8. ^{1}H NMR (600 MHz) data of Fr3-1-3 (in CD_3OD) 10

Figure S9. ^{13}C NMR (151 MHz) data of Fr3-1-3 (in CD_3OD) 11

Figure S10. ESI-MS data of Fr3-1-4 12

Figure S11. ^{1}H NMR (600 MHz) data of Fr3-1-4 (in CD_3OD) 13

Figure S12. ^{13}C NMR (151 MHz) data of Fr3-1-4 (in CD_3OD) 14

Figure S13. Interaction of Fr3-1-1 with active sites of 9 potential target proteins. 15

Figure S14. Interaction of Fr3-1-2 with active sites of 9 potential target proteins. 16

Figure S15. Interaction of Fr3-1-3 with active sites of 9 potential target proteins. 17

Figure S16. Interaction of Fr3-1-4 with active sites of 9 potential target proteins. 18

Figure S1. ESI-MS data of Fr3-1-1

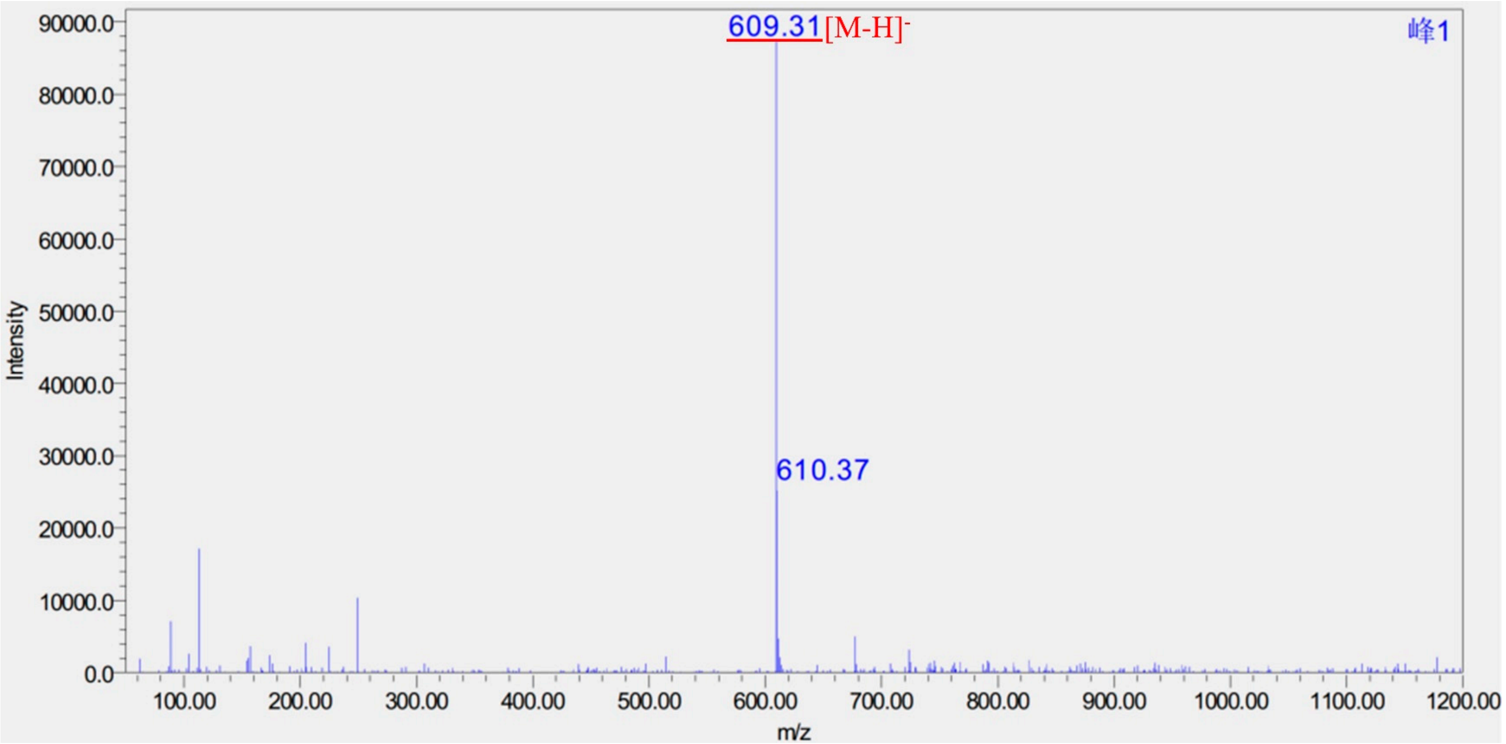

Figure S2. ^{1}H NMR (600 MHz) data of Fr3-1-1 (in CD_3OD)

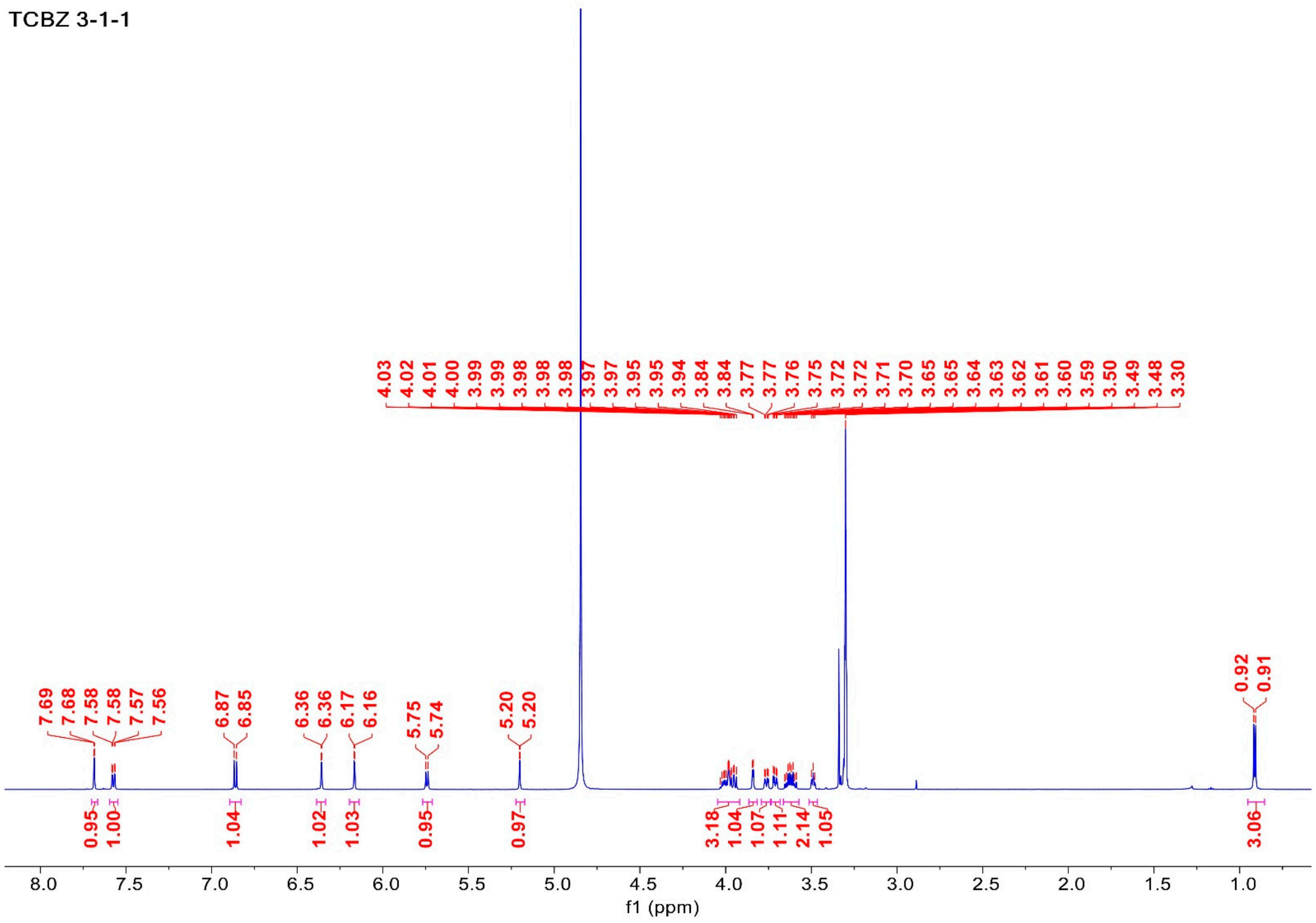

Figure S3. ^{13}C NMR (151 MHz) data of Fr3-1-1 (in CD_3OD)

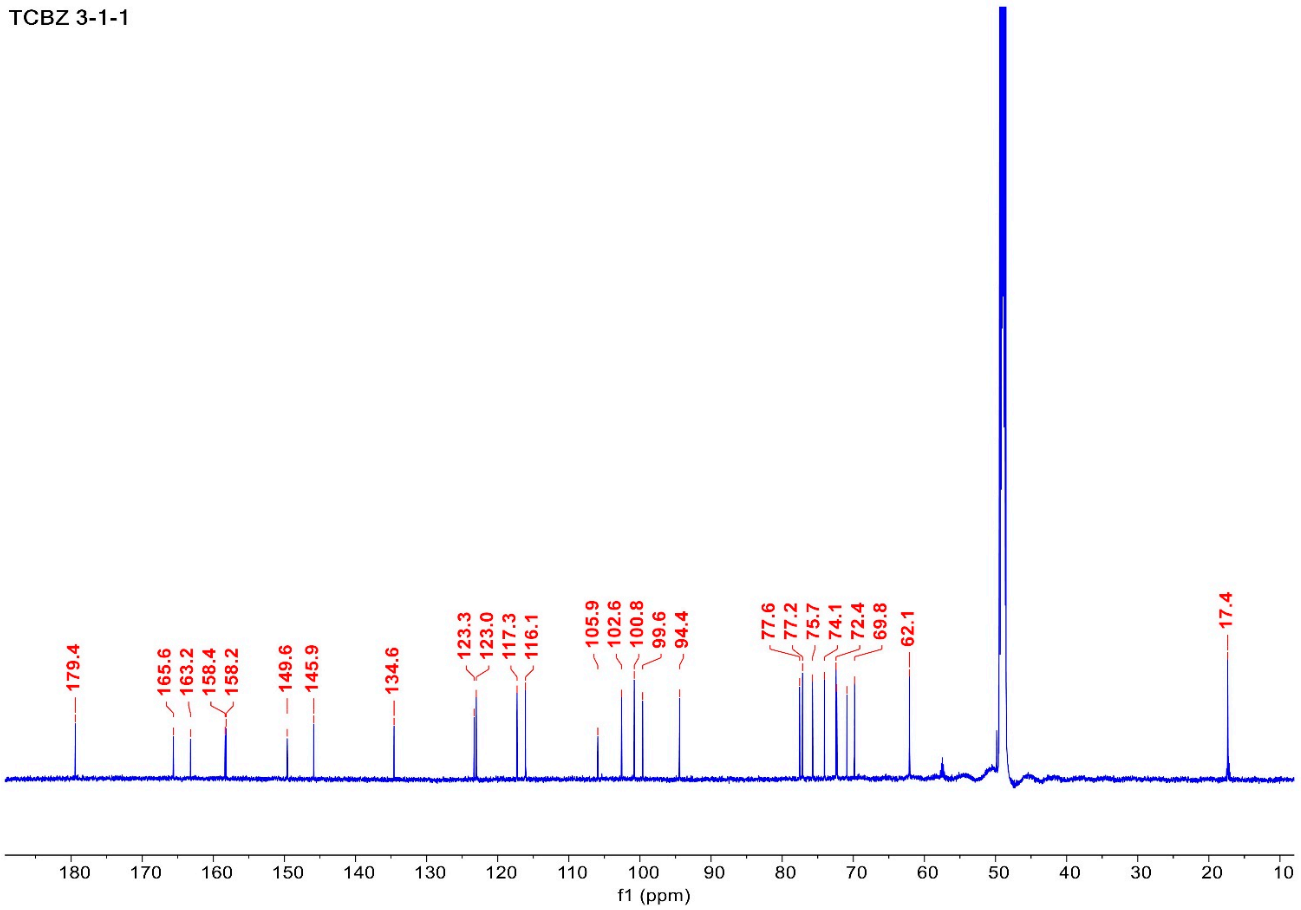

Figure S4. ESI-MS data of Fr3-1-2

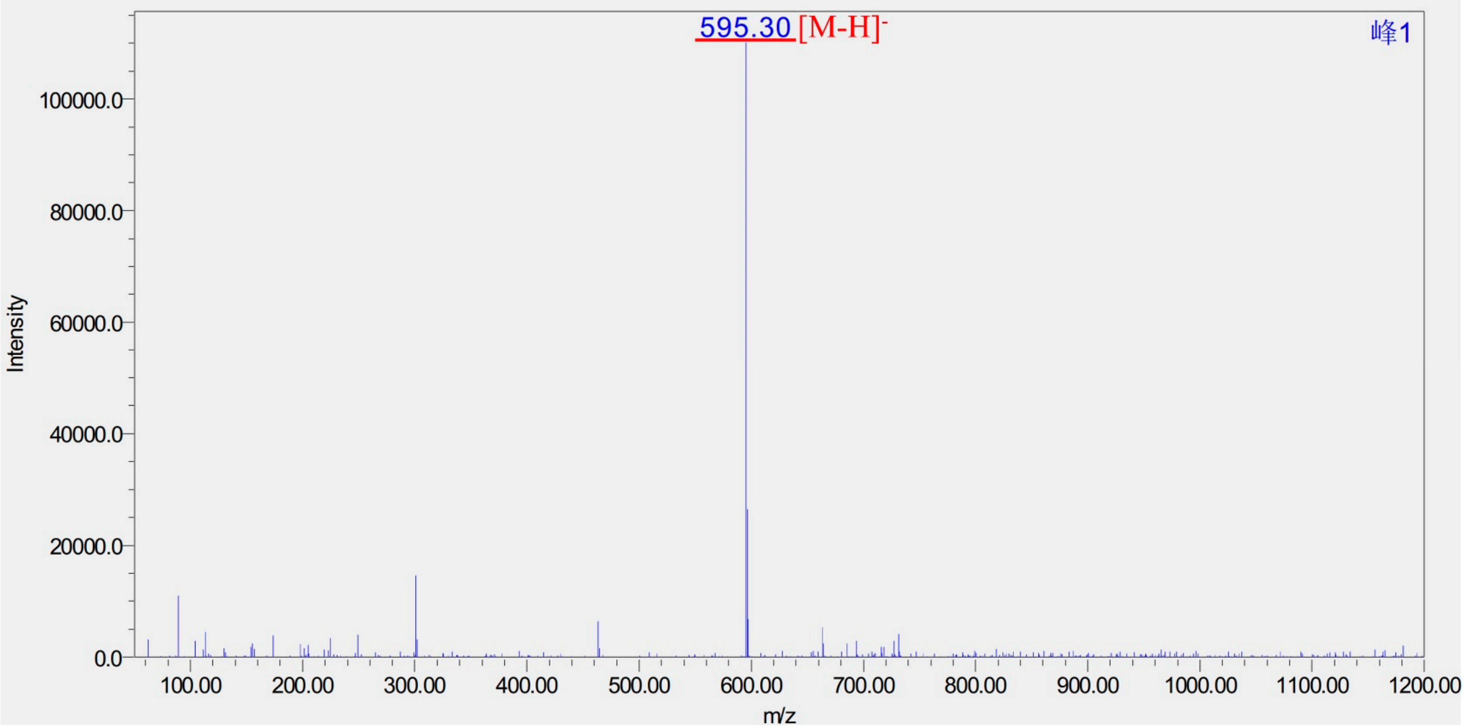

Figure S5. ^{1}H NMR (600 MHz) data of Fr3-1-2 (in CD_3OD)

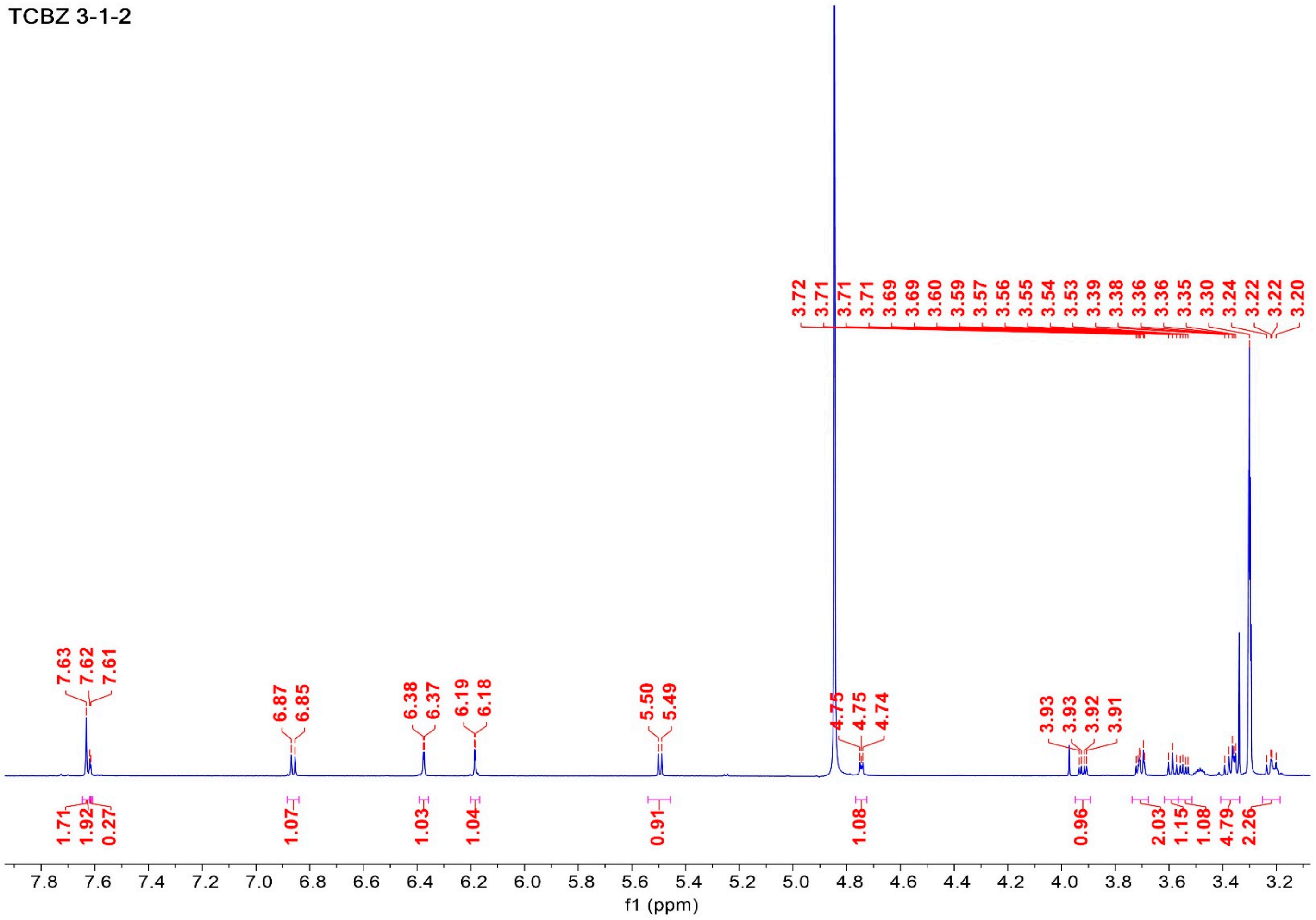

Figure S6. ^{13}C NMR (151 MHz) data of Fr3-1-2 (in CD_3OD)

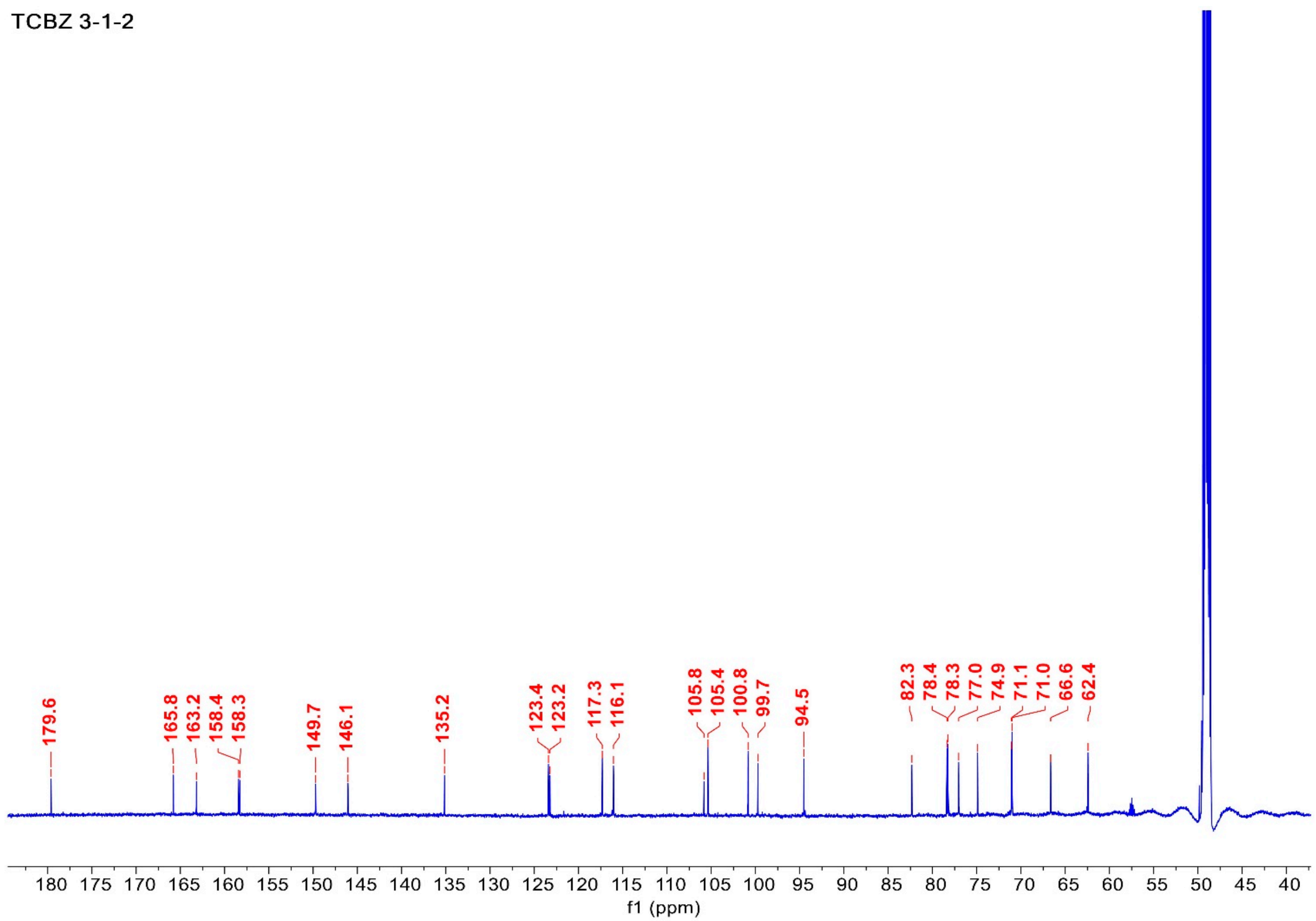

Figure S7. ESI-MS data of Fr3-1-3

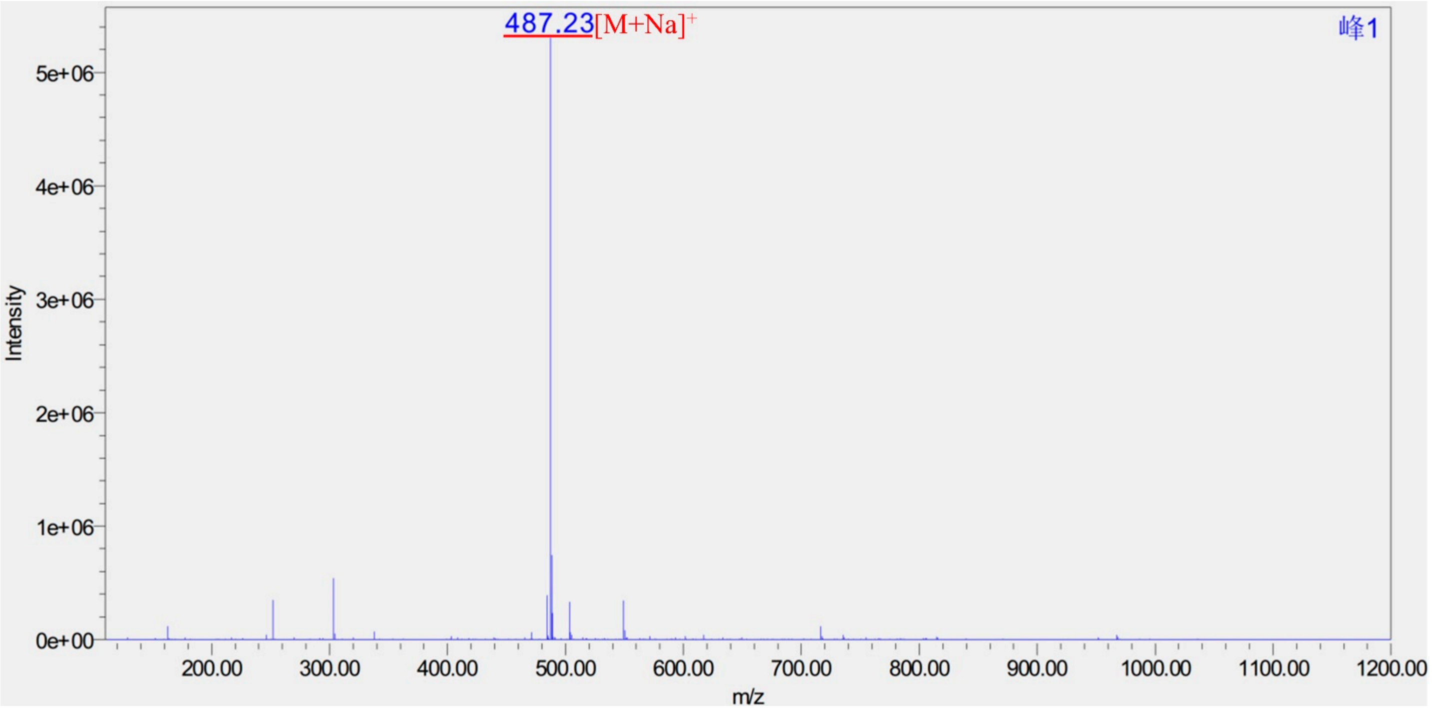

Figure S8. ^{1}H NMR (600 MHz) data of Fr3-1-3 (in CD_3OD)

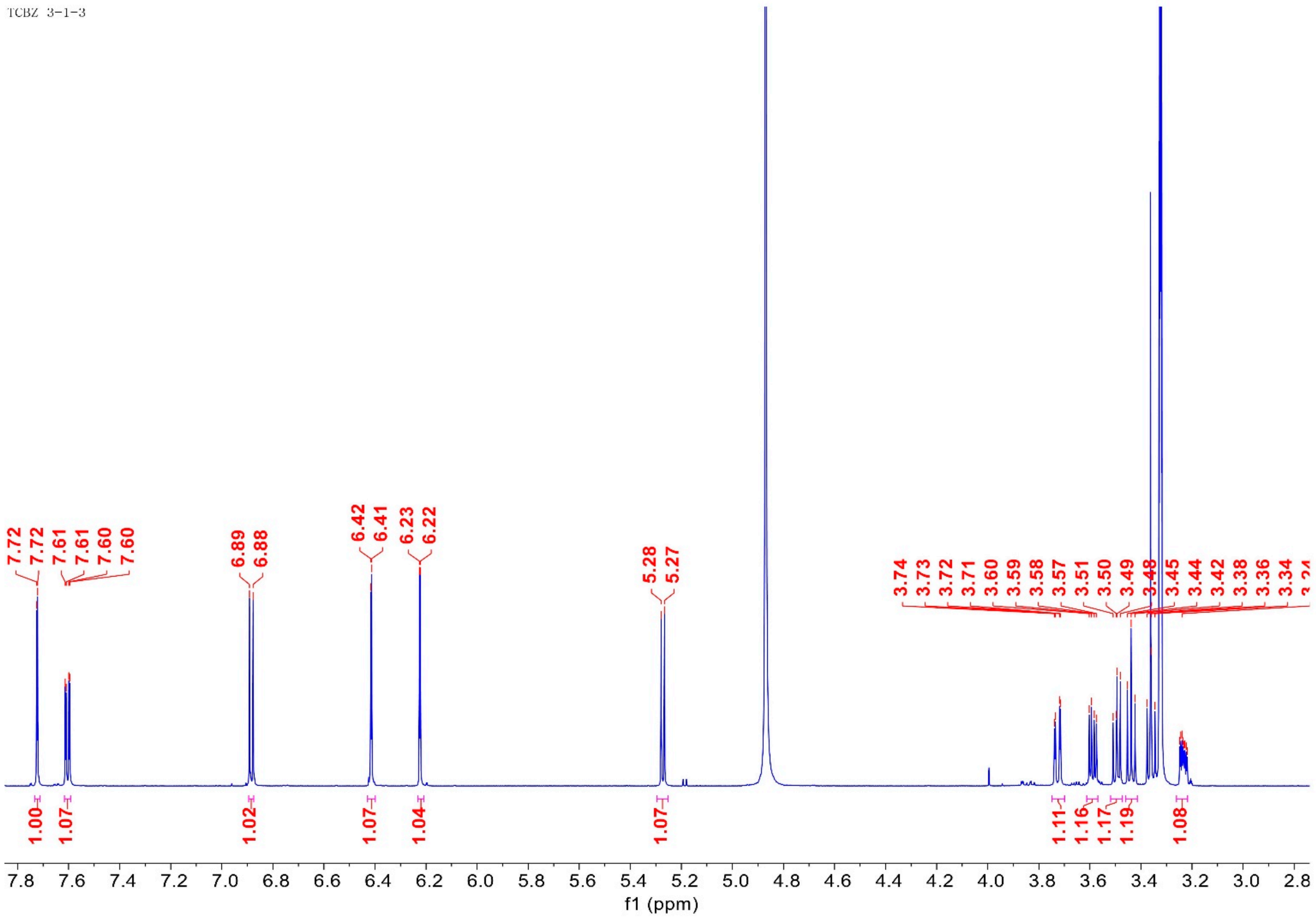

Figure S9. ^{13}C NMR (151 MHz) data of Fr3-1-3 (in CD_3OD)

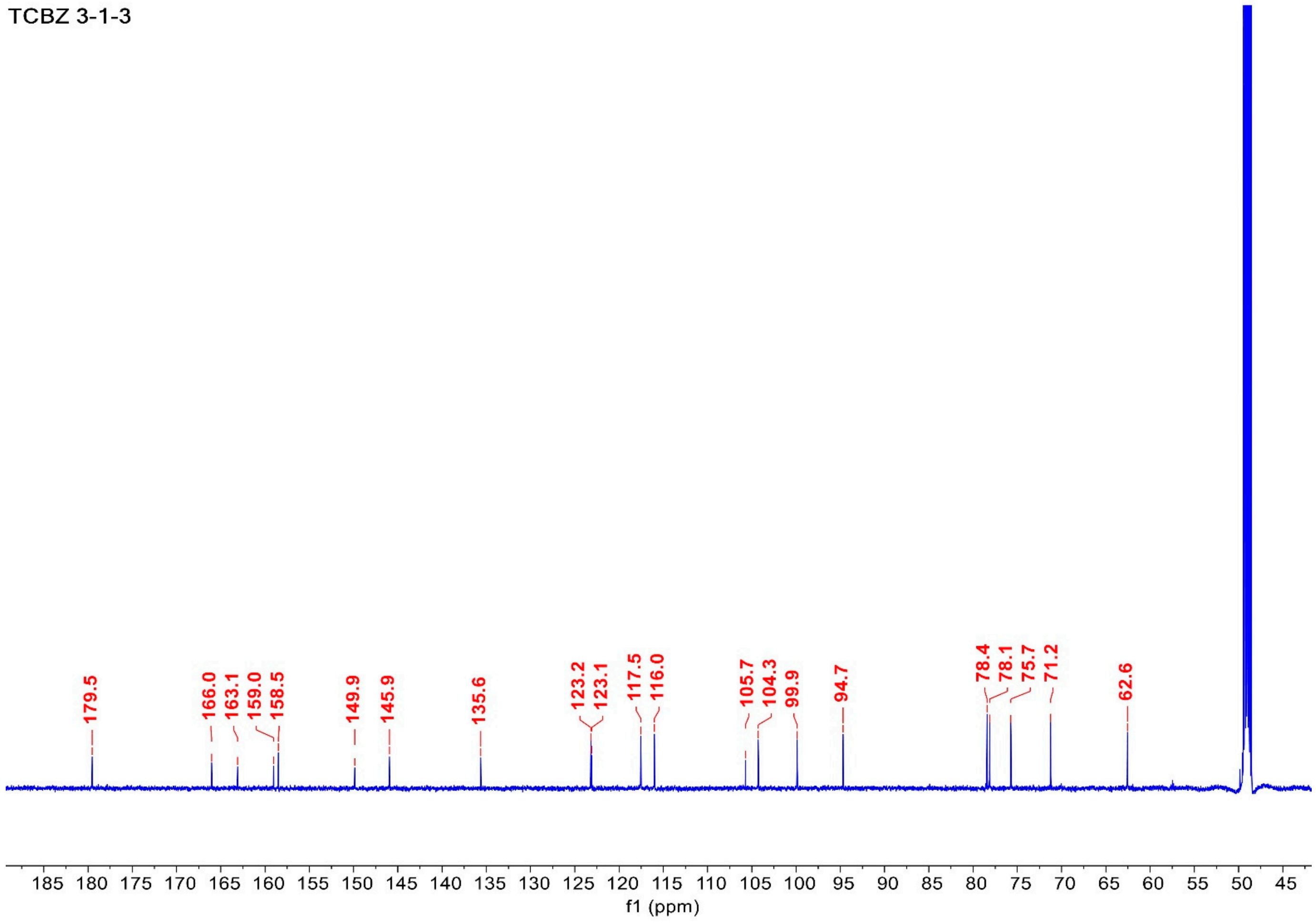

Figure S10. ESI-MS data of Fr3-1-4

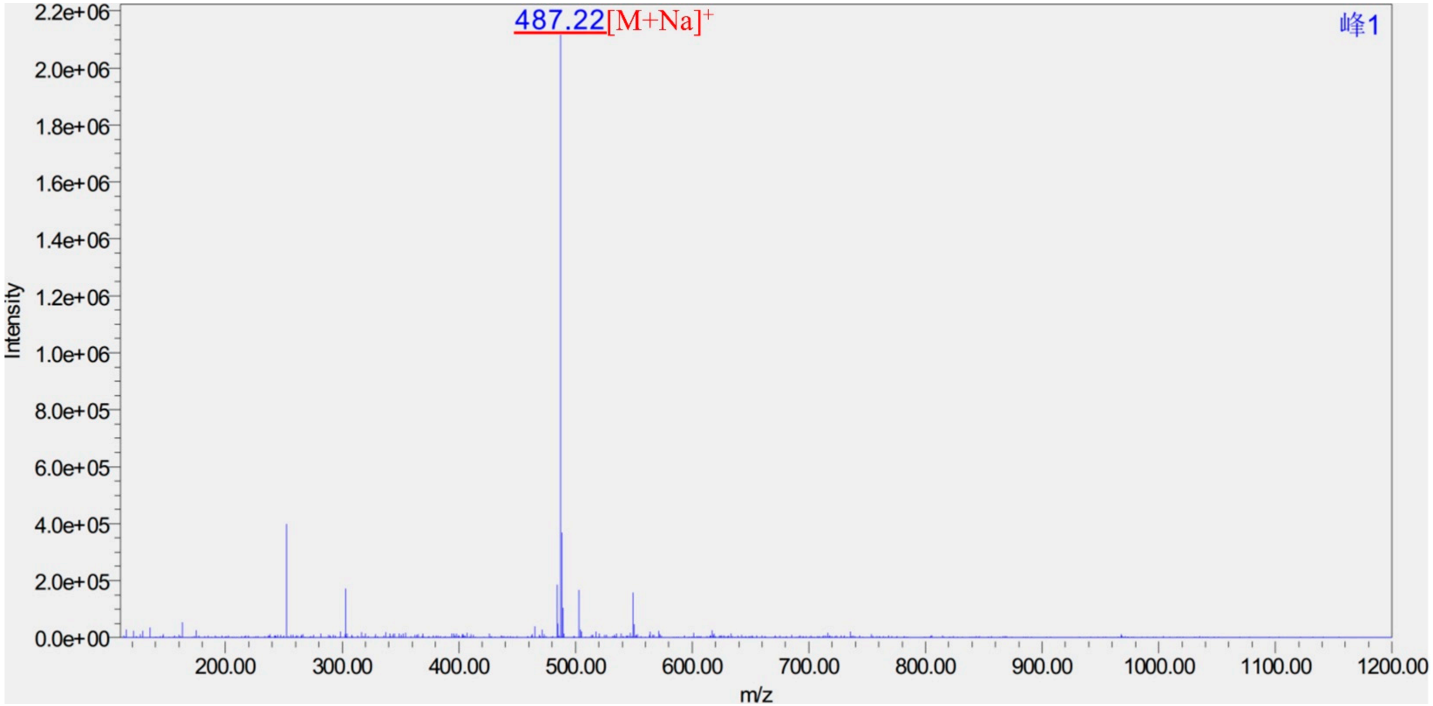

Figure S11. ^{1}H NMR (600 MHz) data of Fr3-1-4 (in CD_3OD)

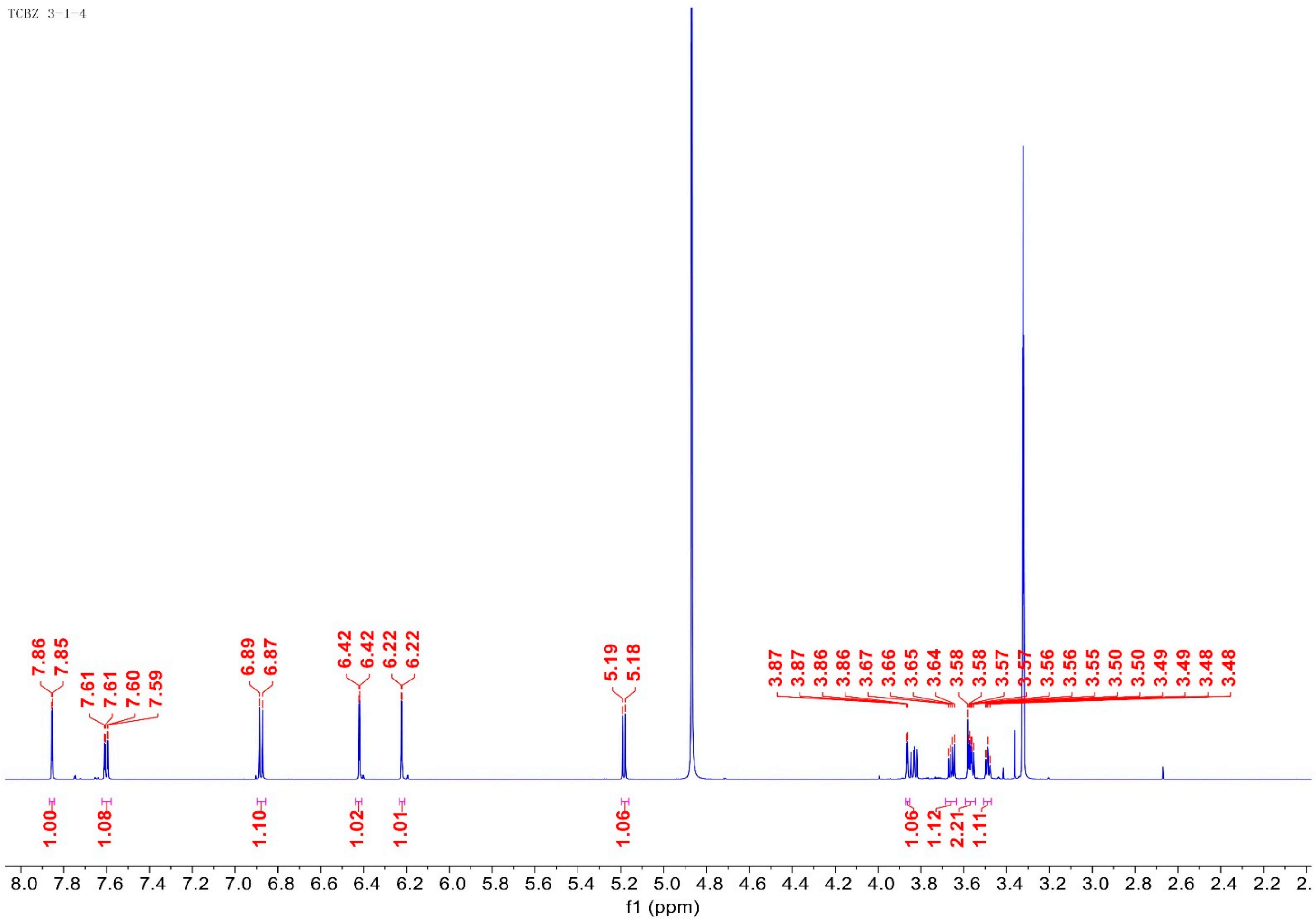

Figure S12. ^{13}C NMR (151 MHz) data of Fr3-1-4 (in CD_3OD)

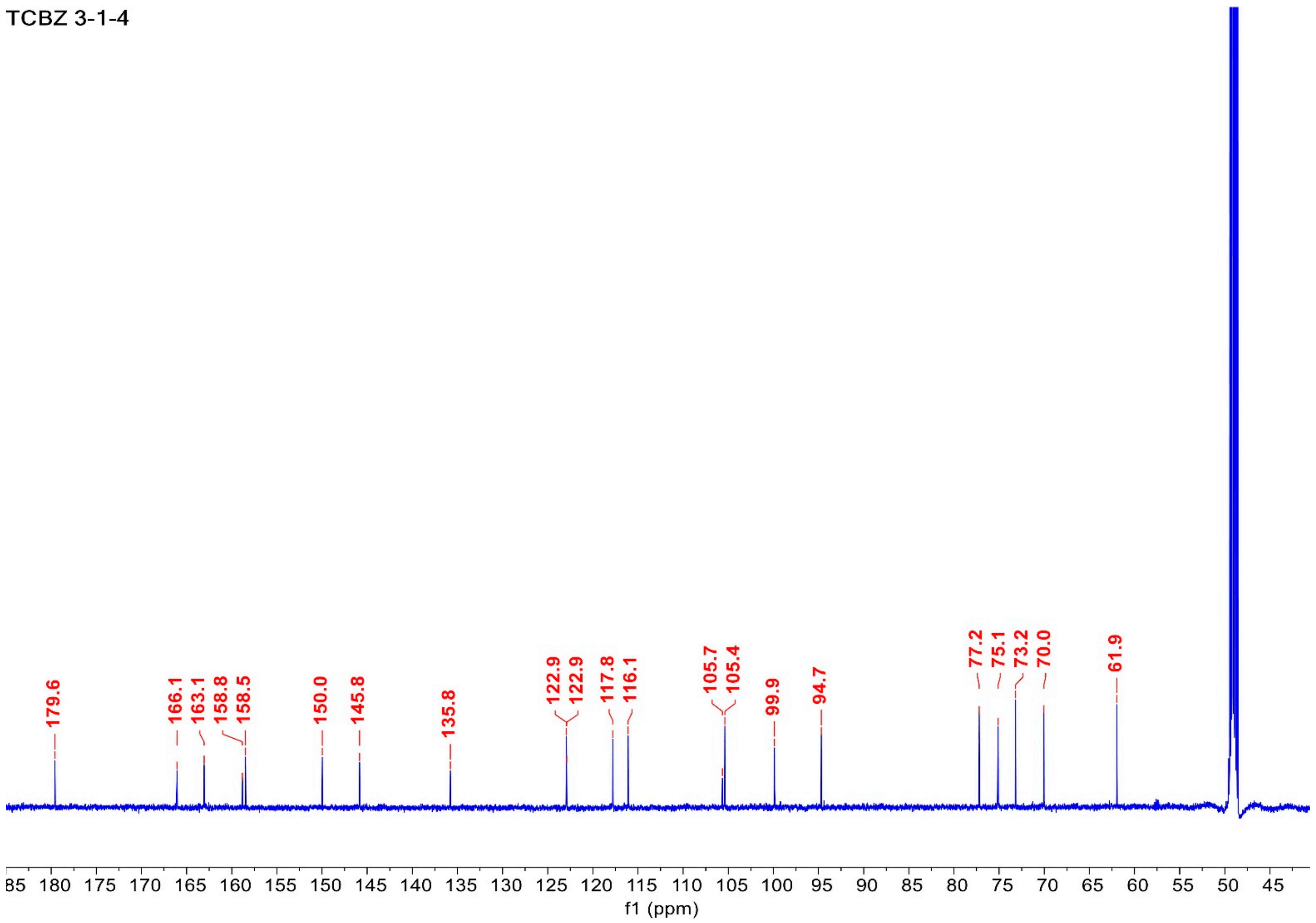

Figure S13. Interaction of Fr3-1-1 with active sites of 9 potential target proteins.

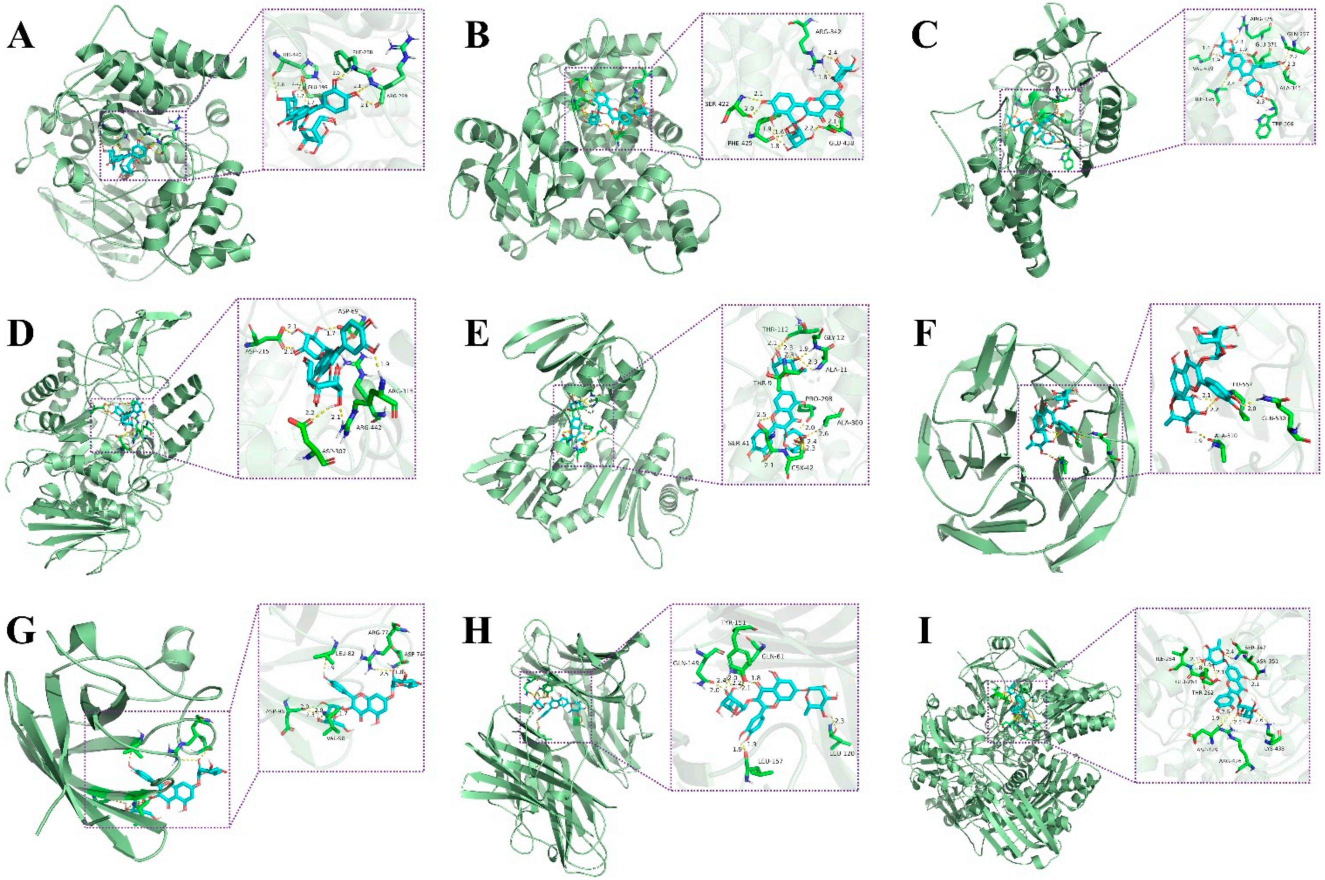

The potential target proteins represented by A-I are: AchE, CYP2C9, iNOS, α-glucosidase, NADPH-oxidase, Nrf2, SOD, TNF-α, XOD, respectively.

Figure S14. Interaction of Fr3-1-2 with active sites of 9 potential target proteins.

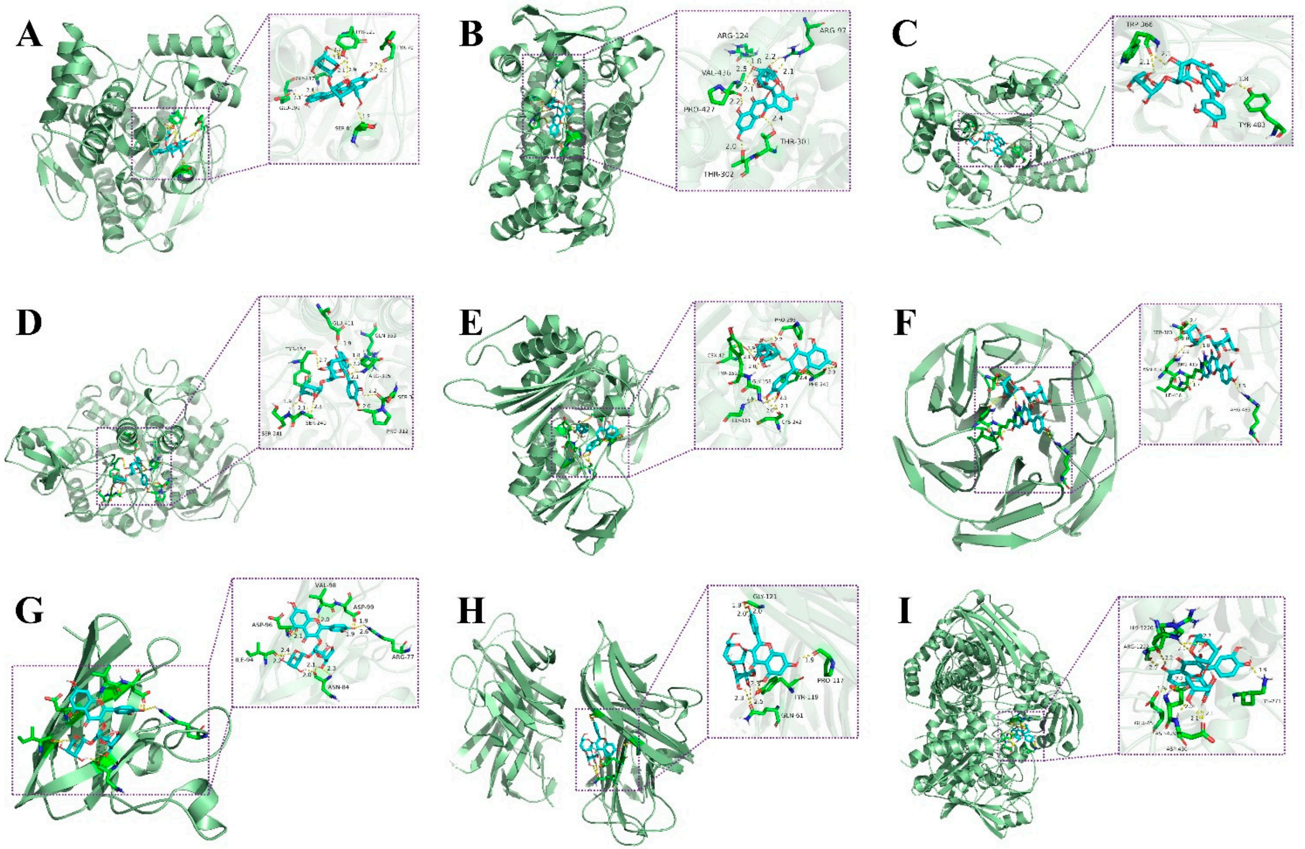

The potential target proteins represented by A-I are: AchE, CYP2C9, iNOS, α-glucosidase, NADPH-oxidase, Nrf2, SOD, TNF-α, XOD, respectively.

Figure S15. Interaction of Fr3-1-3 with active sites of 9 potential target proteins.

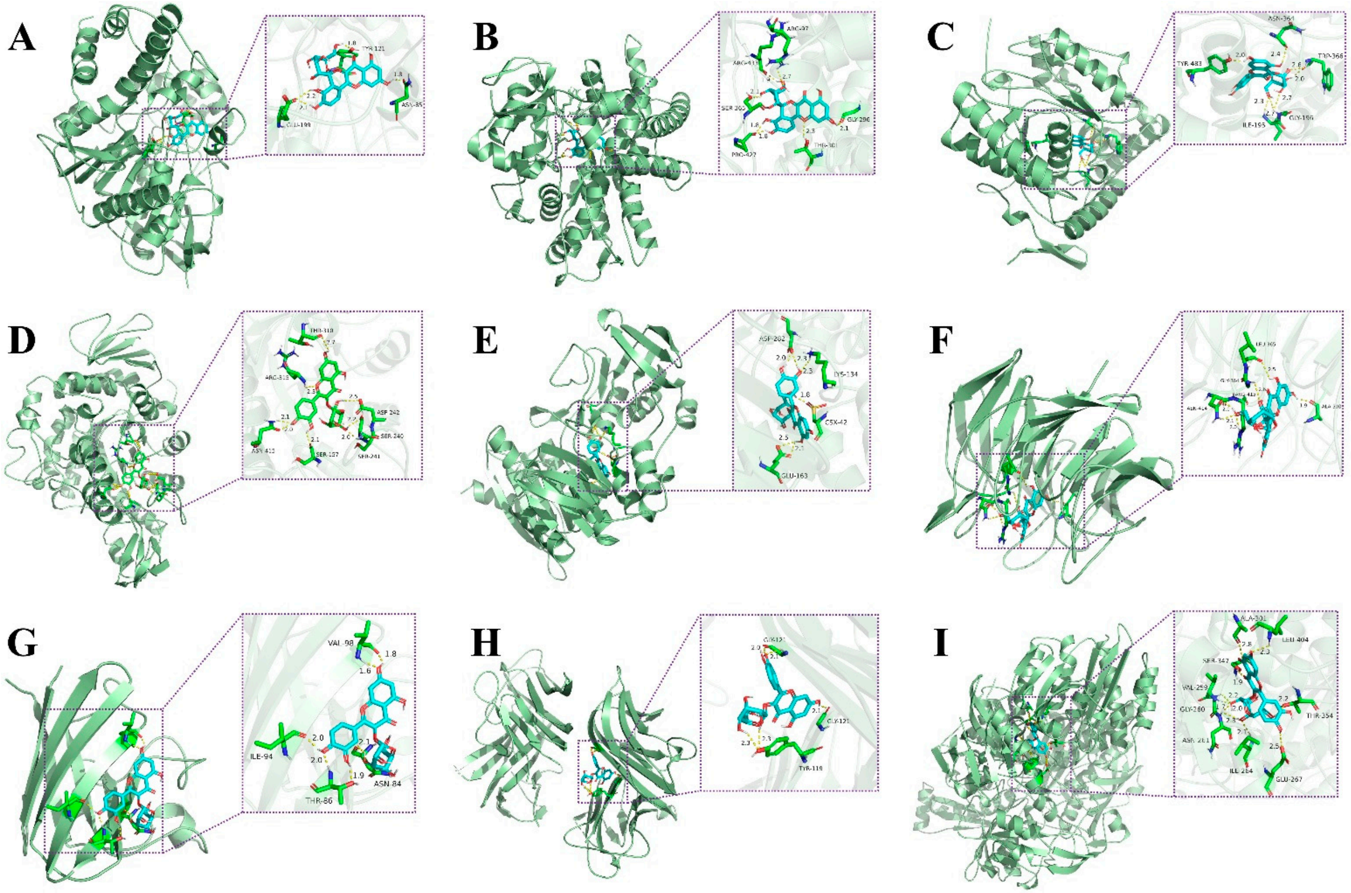

The potential target proteins represented by A-I are: AchE, CYP2C9, iNOS, α-glucosidase, NADPH-oxidase, Nrf2, SOD, TNF-α, XOD, respectively.

Figure S16. Interaction of Fr3-1-4 with active sites of 9 potential target proteins.

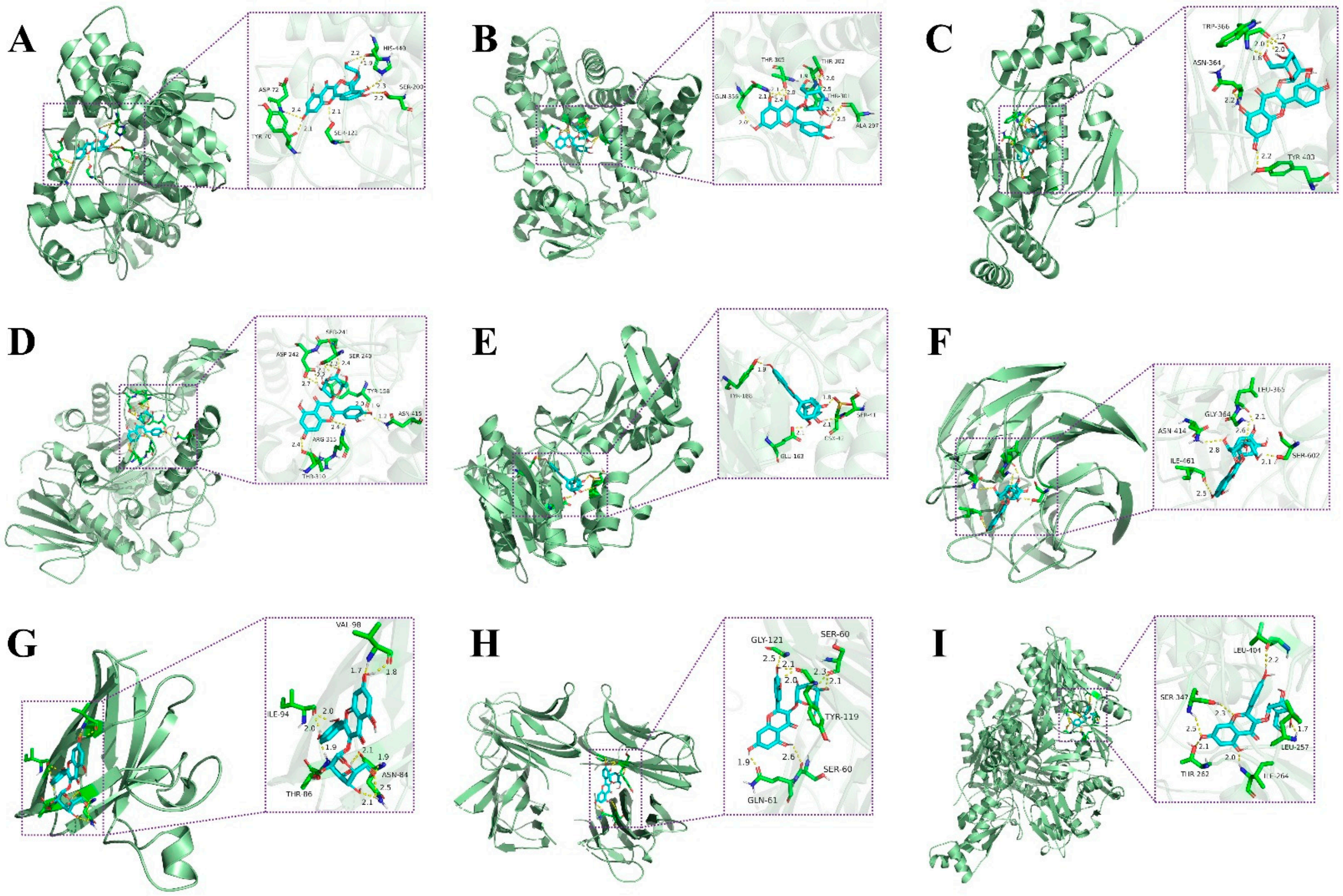

The potential target proteins represented by A-I are: AchE, CYP2C9, iNOS, α-glucosidase, NADPH-oxidase, Nrf2, SOD, TNF-α, XOD, respectively.
